# Enhanced chimp hierarchy optimization algorithm with adaptive lens imaging for feature selection in data classification

**DOI:** 10.1038/s41598-024-57518-9

**Published:** 2024-03-22

**Authors:** Li Zhang, XiaoBo Chen

**Affiliations:** 1 College of Computer Engineering, Jiangsu University of Technology, Changzhou, 213001 People’s Republic of China; 2 Key Laboratory of Symbolic Computation and Knowledge Engineering of Ministry of Education Jilin University, Changchun, 130012 People’s Republic of China; 3 People’s Bank of China Changzhou City Center Branch, Changzhou, 213001 Jiangsu People’s Republic of China

**Keywords:** Feature selection, Chimp optimization algorithm, Hierarchy, Social class factor, Local optimal, Engineering, Mathematics and computing

## Abstract

Feature selection is a critical component of machine learning and data mining to remove redundant and irrelevant features from a dataset. The Chimp Optimization Algorithm (CHoA) is widely applicable to various optimization problems due to its low number of parameters and fast convergence rate. However, CHoA has a weak exploration capability and tends to fall into local optimal solutions in solving the feature selection process, leading to ineffective removal of irrelevant and redundant features. To solve this problem, this paper proposes the Enhanced Chimp Hierarchy Optimization Algorithm for adaptive lens imaging (ALI-CHoASH) for searching the optimal classification problems for the optimal subset of features. Specifically, to enhance the exploration and exploitation capability of CHoA, we designed a chimp social hierarchy. We employed a novel social class factor to label the class situation of each chimp, enabling effective modelling and optimization of the relationships among chimp individuals. Then, to parse chimps’ social and collaborative behaviours with different social classes, we introduce other attacking prey and autonomous search strategies to help chimp individuals approach the optimal solution faster. In addition, considering the poor diversity of chimp groups in the late iteration, we propose an adaptive lens imaging back-learning strategy to avoid the algorithm falling into a local optimum. Finally, we validate the improvement of ALI-CHoASH in exploration and exploitation capabilities using several high-dimensional datasets. We also compare ALI-CHoASH with eight state-of-the-art methods in classification accuracy, feature subset size, and computation time to demonstrate its superiority.

## Introduction

Classification is an essential topic in machine learning. High-dimensional datasets are becoming more and more common as new data collection techniques continue to emerge. However, not all features in a dataset are relevant to the classification goal. It is becoming increasingly important to quickly and accurately select the most valuable features from a large dataset^1^. Feature selection allows the selection of the most useful features from the original high-dimensional data while preserving the physical properties of the original features^2^. Therefore, feature selection is a critical preprocessing step in data mining and machine learning^3,4^.

The search strategies for feature subsets^5,6^ are generally classified into heuristic, random, and complete. However, the arbitrary search cannot avoid repeating the search of already explored solutions due to the lack of memory or learning mechanism, which results in a waste of resources. Meanwhile, the search space grows exponentially as the problem dimension increases, making it impractical to perform a complete search. In contrast, heuristic search^7^ performs well in dealing with complex problems and is valuable for solving optimal or near-optimal solutions. Therefore, metaheuristic algorithms have received much attention and research in recent decades for their efficient performance in solving high-dimensional optimization problems^8^.

Swarm Intelligence^9^ efficiently finds optimal or near-optimal solutions in the solution space through interactions and information sharing among individuals in the search space. As a kind of meta-inspired algorithm, group intelligence algorithms are widely used and show great potential with their applications in optimization, data mining, and machine learning^10^. In the past decades, many swarm intelligences have been proposed. For example, Grey Wolf Optimizer(GWO)^11^, Salp Swarm Algorithm(SSA)^12^, Harris hawks optimization(HHO)^13^, Slime Mould algorithm(SMA)^14^, Bald Eagle Search optimization algorithm(BES)^15^, Sand Cat swarm optimization(SCSO)^16^. These algorithms effectively solve multi-objective optimization problems and approximate the true Pareto optimal solution^17,18^. However, these swarm intelligence algorithms have a common disadvantage: they may converge slowly during the search process and easily fall into local optimal solutions^19^. The main reason for these drawbacks is the imbalance between exploration and exploitation throughout the search process^20^.

The chimp optimization algorithm (CHoA)^21^ is a swarm intelligence optimization algorithm proposed by Khishe et al. in 2020, inspired by the chimp population’s hierarchy and hunting behaviour. It uses mathematical models to simulate the optimal behaviour of chimp populations through herding, chasing, and attacking to achieve predation. ChOA has the advantages of being simple in principle, requiring few tuning parameters, and being accessible to implement. These advantages of ChOA have motivated many researchers to apply it to many practical engineering tasks. For example, breast cancer diagnosis^22^, photovoltaic and solar cell performance optimization^23^, and Internet of Things applications^24^. However, like swarm intelligence algorithms, such as the classical butterfly optimization algorithm^25^, ChOA suffers from some shortcomings, such as slow convergence, low optimization search accuracy, and a tendency to fall into local optimality. These problems are especially prominent for complex high-dimensional optimization problems with multiple local extremes. The main reason for this phenomenon is attributed to the fact that the hunting mechanism of the basic CHoA is based on the information of the globally optimal attackers, barrier, chaser, and driver, which leads to the fact that the basic CHoA will gradually lose its exploration and exploitation capability in the later optimization stages. To solve this problem, researchers have proposed many variants of CHoA to compensate for this deficiency. For example, Kaur et al. proposed the SChoA algorithm^26^, in which the sine-cosine function updates the chimp’s equations to improve the convergence speed of the algorithm and find the global minimum. Jia et al. proposed the EChOA algorithm^27^, which enhances the exploration and exploitation capabilities of the original ChOA algorithm through polynomial variation, Pearman rank correlation coefficients, and beetle tentacle operators. Wang et al. proposed the DLFChOA algorithm^28^, which smoothly transitions the search agent from the exploration phase to the exploitation phase by introducing a dynamic Lévy flight technique. This technique helps to increase the diversity of the algorithm in some complex issues and avoid the stagnation phenomenon of falling into local optimal solutions. However, the adaptive trade-off between exploration and exploitation is not considered in these different CHoA variants.

Therefore, we propose an enhanced chimp hierarchy optimization algorithm for adaptive lens imaging (ALI-CHoASH). The ALI-CHoASH algorithm introduces three innovations to improve the performance of CHoA:

- A chimp social hierarchy was designed to enhance CHoA exploration and exploitation by tagging individual chimps with a social class factor to enable modelling and optimizing inter-individual relationships.
- Parsing chimps’ social and collaborative behaviours from different social classes. Introducing different prey-attacking strategies and autonomous searching strategies in each social class, the approach can fully reflect the leading role of high-ranking chimps to lower-ranking chimps and fully exploit the independent mobility of individual chimpanzees to improve the diversity of the population.
- In the late iteration of the algorithm, an opposite learning strategy with adaptive lens imaging is proposed, which expands the algorithm’s global exploitation capability and improves the population’s diversity, thus preventing the algorithm from falling into the local optimal solution.

In summary, the ALI-CHoASH algorithm improves the performance of CHoA by introducing the chimp social hierarchy, different strategies for attacking prey and autonomous searching strategies, and an oppositional learning strategy for adaptive lens imaging, which enhances the exploration and exploitation of feature selection, thus preventing from falling into local optima. To verify its effectiveness in feature selection, extensive experiments are conducted to compare the ALI-CHoASH algorithm with the CHoA^21^, SChoA^26^, GMPBSA^29^, GWO^11^, SSA^12^ HHO^13^, SMA^14^, BES^15^ algorithms, respectively. ALI-CHoASH is more effective in classification accuracy average and optimal fitness values.

The remainder of this work is summarized in the following structure. “Related work” Section describes related work on existing ChoA variants. “Background” Section briefly describes and introduces the basic CHoA algorithm and the convex lens imaging principle. “Enhanced chimp hierarchy optimization algorithm for adaptive lens imaging” Section presents our proposed ALI-CHoASH algorithm for feature selection. In “Experimental analyses and discussions” Section, a series of experiments are performed and the results are discussed in detail. Finally, “Conclusion” Section is drawn, and the following research directions are given.

## Related work

Exploration and exploitation are integral in swarm intelligence optimization algorithms^30,31^. Exploration provides global search capabilities that help algorithms discover potential solutions. Conversely, exploitation improves the quality and accuracy of solutions through local search and optimization. Therefore, the main challenge of intelligent optimization algorithms is finding the best balance between exploration and exploitation, maintaining diversity in the solution space, and preventing the algorithms from prematurely converging to local optimal solutions. So far, scholars have made many improvements to enhance the performance of intelligent optimization algorithms. According to the literature review, improvements to intelligent optimization algorithms can be classified into the following three categories.

### Feature selection models are built using intelligent optimization algorithms fused with binary conversion functions

For example, Khosrav et al.^32^ proposed BGTOAV and BGTOAS for feature selection, which can improve the performance of binary group teaching optimization algorithms by introducing improvements such as local search, chaotic mapping, new binary operators, and oppositional learning strategies to solve high-dimensional feature selection problems. Pashaei et al.^33^ proposed an orangutan optimization algorithm-based Packed feature selection method, which introduces two binary variants of the orangutan optimization algorithm to solve the classification of biomedical data. Experiments demonstrate the method’s effectiveness in feature selection and classification accuracy, and it outperforms other wrapper-based feature selection methods and filter-based feature selection methods on multiple datasets. This provides an effective algorithm and an improved method for solving the biomedical data classification problem. Guha et al.^34^ proposed the DEOSA algorithm for feature selection, which first maps the continuous values of the EO (Equilibrium Optimizer)^35^ to the binary domain by using a U-shape transformation function. Then, Simulated Annealing (SA) is introduced to enhance the local exploitation capability of the DEOSA algorithm. Zhuang et al.^36^ proposed the PBAOA algorithm for feature selection. In the PBAOA algorithm, multiplication and division operators are first utilized for exploring the solution space, while subtraction and addition operators are used to develop existing solutions. Then, four types of transformation functions are used to improve the robustness and adaptability of the PBAOA algorithm, speed up the convergence and search efficiency of the algorithm, and improve the algorithm’s performance. Fatahi et al. proposed an Improved Binary Quantum-based Avian Navigation Optimizer Algorithm (IBQANA)^37^, which solves the problem of binary versions of meta-heuristic algorithms that produce sub-optimal solutions. Nadimi-Shahraki et al. proposed a new binary starling murmuration optimizer (BSMO)^38^, which solves complex engineering problems and finds the optimal subset of features. Nadimi-Shahraki et al. proposed Binary Approaches of Quantum-Based Avian Navigation Optimizer (BQANA)^39^. This algorithm exploits the scalability of QANA to efficiently select the optimal subset of features from a high-dimensional medical dataset using two different approaches.

### Improve the search mechanism to enhance the algorithm’s performance

For example, Mostafa et al.^40^ proposed an improved chameleon population algorithm (mCSA) for feature selection. mCSA improves the performance of the algorithm by three improvements such as introducing a nonlinear transfer operator, randomizing the Lévy flight control parameter, and borrowing the depletion mechanism from artificial ecosystems optimization algorithms. Long et al.^41^ proposed the VBOA algorithm, which firstly improved the algorithm’s performance by introducing velocity and memory terms and designed an improved position update equation for BOA. Then, a refraction-based learning strategy was introduced into the butterfly optimization algorithm to enhance diversity and exploration. Finally, experimental results demonstrate the effectiveness of the VBOA algorithm for high-dimensional optimization problems. Saffari et al.^42^ proposed the fuzzy-chOA algorithm, which uses fuzzy logic to adjust the control parameters of the ChOA and applies this method to change the relationship between the exploration and exploitation phases. Houssein et al.^43^ introduced the mSTOA algorithm, which employs a balanced exploration/exploitation strategy, an adaptive control parameter strategy, and a population reduction strategy to improve the STOA algorithm’s tendency to fall into suboptimal solutions when solving the feature selection problem. Chhabra et al. introduced an improved Bald Eagle Search (mBES) algorithm^44^, which aims to solve the original BES algorithm’s insufficient searching issues efficiency and tendency to fall into local optimums. mBES is a new algorithm for the exploration of a large area of a large area of a river. To fall into local optima. mBES algorithm is improved by introducing three improvements. Firstly, the positions of individual solutions are updated using oppositional learning to enhance the exploration capability. Secondly, Chaotic Local Search is used to improve the local search capability of the algorithm. Finally, Transition and phasor operators balance the relationship between exploration and exploitation. Khishe et al.^45^ proposed an improved orangutan optimization algorithm (OBLChOA), which improves the exploration and exploitation capabilities of ChOA by introducing greedy search and oppositional learning (OBL)-based methods. These improvements aim to address the slow convergence speed and lack of exploration capability of ChOA. Xu et al.^46^ study demonstrated the effectiveness of the Enhanced Grasshopper Optimization Algorithm (EGOA) in solving single-objective optimization problems. By introducing elite oppositional learning and a simplified Gaussian strategy, EGOA can discover solutions better at an early stage while having good search agent update capability. For solving globally constrained and unconstrained optimization problems and feature selection problems, EGOA exhibits good robustness and performance. This provides valuable tools and methods for optimization and feature selection in real-world situations. Bo et al.^47^ proposed an Evolutionary Orangutan Optimization Algorithm (GSOBL-ChOA), which utilizes Greedy Search and Oppositional Learning to increase the exploration and exploitation capabilities of ChOA in solving real-world engineering-constrained problems, respectively. Nadimi-Shahraki et al. proposed the Enhanced whale optimization algorithm (E-WOA)^48^, which uses three effective search strategies named migrating, preferential selecting, and enriched encircling prey, effectively solving the global optimization problem and improving the efficiency of feature selection.

### Incorporates different algorithms to improve the performance of the algorithm

Gong et al.^49^ This paper proposed an improved Orangutan Optimization Algorithm (NChOA) by embedding a clustering technique that allows it to handle various local/global optimal solutions better and retain the values of these optimal solutions until termination. This method combines the individual optimal algorithmic features of Particle Swarm Optimization (PSO) and local search techniques. Pasandideh et al.^50^ proposed a Sine Cosine Orangutan Optimization Algorithm (SChoA) combining the Sine Cosine Function and ChoA as well as combining ChoA with Particle Swarm Optimization (PSO) to form the ChoA-PSO algorithm. Finally, these new meta-heuristic algorithms are combined to solve the problem of optimal operation strategies for dam reservoirs. Kumari proposed^51^ improved variants of ChoA. One of these variants combines the existing ChoA with the SHO algorithm to enhance the exploration phase of the existing ChoA, named IChoA-SHO. The other variant aims to improve the exploitation search capability of the existing ChoA. These improved variants aim to solve the problem of poor convergence and the tendency to fall into local minima of traditional orangutan optimization algorithms for high-dimensional problems.

In summary, selecting appropriate search mechanisms is crucial for improving intelligent optimization algorithms. Therefore, the focus of this study is to propose the enhancement of the chimpanzee social hierarchy to achieve effective modelling and optimization of inter-individual relationships among chimps and to improve the exploration and exploitation capabilities of the underlying CHoA to prevent from falling into a locally optimal solution by introducing different strategies for attacking the prey, an autonomous searching strategy as well as an oppositional learning strategy with adaptive lens imaging, which is also applied to the feature selection problem.

## Background

### Chimp optimization algorithm

**Figure 1:**
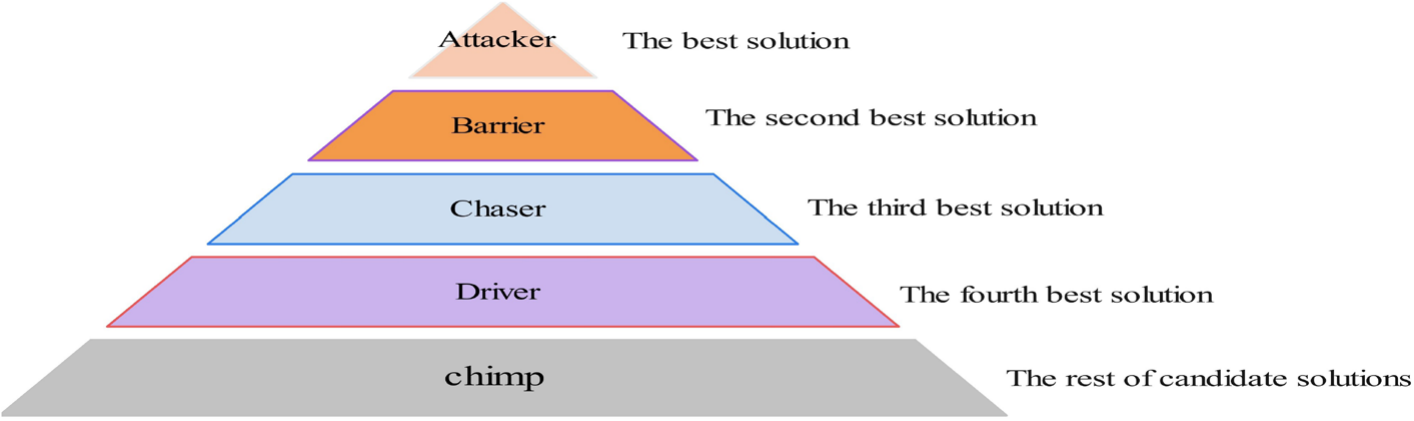
Hierarchical diagram of the chimp optimization algorithm.

Chimps live in groups with a strict hierarchy among them. The chimp family is divided into five classes: attackers, barriers, chasers, drivers and common chimps. As shown in Fig. 1, the attacker chimp is located at the top of the social hierarchy and is the supreme ruler and manager of the chimp group. The barrier chimp is found at the second level, equivalent to the deputy leader in the chimp group and is responsible for taking over the leadership from the attacker chimp. The chaser chimps are located in the third tier and are subservient to both attackers and barriers. The driver chimps are found in the fourth tier and are subordinate to the attackers, barriers, and chasers but can rule over the common chimps. The common chimp is located at the bottom of the hierarchy and always has to obey other chimps of higher status.

In the CHoA algorithm, the chimp group in the search space mainly uses the four best-performing chimps to guide the other chimps to search towards their optimal area, while in the continuous iterative search process, the four chimps, namely the attacker, the barrier, the chaser and the driver, predict the possible location of the captured object, i.e., by guiding the continuous search for the global optimal solution. Thus, the mathematical model of a chimp chasing prey during the search process is as follows:

1 $$\begin{aligned} {X_{chimp}}\left( {t + 1} \right) = {X_{prey}}\left( t \right) - a \cdot \left| {C \cdot {X_{prey}}\left( t \right) - m \cdot {X_{chimp}}\left( t \right) } \right| \end{aligned}$$

In Eq. (1), ${X_{prey}}$the position vector of the prey, ${X_{chimp}}$ the position vector of the current individual chimp, *t* the number of current iterations, and *a*, *C*, *m* the coefficient vector, which is calculated as follows:

2 $$\begin{aligned}{} & {} a = 2 \cdot f \cdot {r_1} - f \end{aligned}$$

3 $$\begin{aligned}{} & {} C = 2 \cdot {r_2} \end{aligned}$$

4 $$\begin{aligned}{} & {} m = Chaotic\_value \end{aligned}$$

5 $$\begin{aligned}{} & {} f = 2.5 - \frac{{2.5 \cdot t}}{T} \end{aligned}$$

Among them, ${r_1}$ and ${r_2}$ are random numbers between $\left[ {0,1} \right]$, respectively. *f* is the convergence factor whose value decreases non-linearly from 2.5 to 0 as the number of iterations increases. *T* is denoted as the maximum number of iterations. *a* is a random vector that determines the distance between the chimp and the prey, with a random number of values between $\left[ { - f,f} \right]$ .*m* is the chaotic vector generated by the chaotic mapping. *C* is the control coefficient for the Chimp expulsion and prey chasing, and its value is a random number between $\left[ {0,2} \right]$.

It is assumed below that in each iteration, the attacker, attacker, barrier, and driver store the four best positions obtained so far, and the remaining chimps need to update their positions based on the positions of the attacker, attacker, barrier, and driver. The following mathematical formula illustrates the process.

6 $$\begin{aligned}{} & {} {d_{attacker }} = \left| {{C_1} \cdot {X_{attacker }} - {m_1} \cdot X\left( t \right) } \right| \end{aligned}$$

7 $$\begin{aligned}{} & {} {d_{barrier}} = \left| {{C_2} \cdot {X_{barrier}} - {m_2} \cdot X\left( t \right) } \right| \end{aligned}$$

8 $$\begin{aligned}{} & {} {d_{chaser}} = \left| {{C_3} \cdot {X_{chaser}} - {m_3} \cdot X\left( t \right) } \right| \end{aligned}$$

9 $$\begin{aligned}{} & {} {d_{driver}} = \left| {{C_4} \cdot {X_{driver}} - {m_4} \cdot X\left( t \right) } \right| \end{aligned}$$

The mathematical model of a chimp attacking its prey is as follows:

10 $$\begin{aligned} {X_1}= & {} {X_{attacker }} - {a_1} \cdot {d_{attacker}} \end{aligned}$$

11 $$\begin{aligned} {X_2}= & {} {X_{barrier}} - {a_2} \cdot {d_{barrier}} \end{aligned}$$

12 $$\begin{aligned} {X_3}= & {} {X_{chaser}} - {a_3} \cdot {d_{chaser}} \end{aligned}$$

13 $$\begin{aligned} {X_4}= & {} {X_{driver}} - {a_4} \cdot {d_{driver}} \end{aligned}$$

14 $$\begin{aligned} {X_{chimp}}\left( {t + 1} \right)= & {} {{\left( {{X_1} + {X_2} + {X_3} + {X_4}} \right) } / 4} \end{aligned}$$

15 $$\begin{aligned} {X_{chimp}}\left( {t + 1} \right)= & {} \left\{ {\begin{array}{*{20}{c}} {Eq.\left( {4} \right) ,\mu < 0.5}\\ {Chaotic\_value,\mu \ge 0.5} \end{array}} \right. \end{aligned}$$

From Eqs. (10)–(15), $X\left( t \right)$ is the position vector of the current Chimp, ${X_{attacker }}$ is the position vector of the attacker, ${X_{barrier}}$ is the position vector of the barrier, ${X_{chaser}}$ is the position vector of the chaser, ${X_{driver}}$ is the position vector of the driver is the updated position vector of the current Chimp. ${X_{chimp}}\left( {t + 1} \right)$ is the chaotic mapping, which is used to update the position of the solution. $Chaotic\_value$ is the chaotic mapping, which is used to update the position of the solution. Eq. (15) shows that the four best individual Chimps estimate the unique Chimp positions while the other chimp updates their positions randomly.

### Principle of convex lens imaging

The rule of convex lens imaging^52^ is an optical principle stating that when an object is out of focus, it will produce an actual inverted image on the opposite side of a convex lens. Figure 3 illustrates this principle.

**Figure 2:**
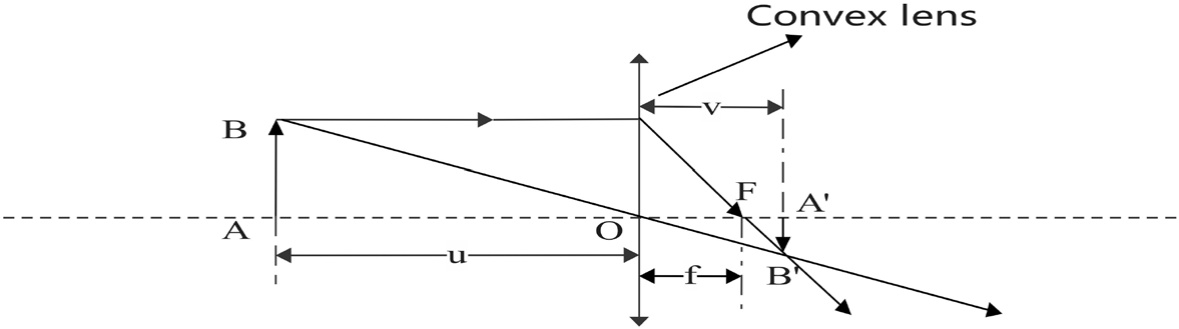
Principle of lens imaging.

The equation for imaging a lens can be derived from Fig. 2 as follows.

16 $$\begin{aligned} \frac{1}{u} + \frac{1}{v} = \frac{1}{f} \end{aligned}$$

*u* is the object distance, *v* is the image distance, and *f* is the lens’s focal length.

## Enhanced chimp hierarchy optimization algorithm for adaptive lens imaging

### Chimp social class operator design and implementation

#### Chimp social hierarchy design ideas

From Eq. (14), it can be seen that when the CHoA algorithm performs an optimization task, all chimps adopt a search strategy with similar behaviours, which may lead to a decrease in the ability of the chimpanzee population to exploit locally. Once the attackers, barriers, chasers and drivers fall into the local optimum, it is difficult for the whole population to escape from the local optimal solution. Therefore, enriching the search strategy of the CHoA algorithm is an effective method that can enhance the algorithm’s global search ability. Currently, the grouping strategy is a common mechanism for multiple search strategies. For example, GTOA (teaching optimization algorithm)^53^ and SO (Snake Optimizer)^54^. The experimental results proved that the grouping strategy using this variety of clusters is very effective. However, there are some drawbacks to the grouping strategy of these algorithms, as follows:

- In the optimization algorithm, the introduction of multiple population strategies and the management of communication and collaboration among them increase the structural complexity of the algorithm.
- The multiple population search strategy requires data communication and information sharing among different populations, which involves a large amount of data communication overhead. Especially when the population size is large and frequent communication is required, the communication overhead will become high, affecting the operation efficiency of the algorithm.
- Parameters such as the number and size of multiple populations and communication strategies are usually required to be set in various population search strategies. The selection of these parameters significantly impacts the algorithm’s performance, and tuning these parameters is also a complex process. To improve the above grouping strategies to enhance the local exploitation of CHoA algorithms. Inspired by the hierarchy in sociological theory, this paper designs a multi-learning strategy for the social hierarchy of the chimp population (CHoASH) to solve the problem of population diversity reduction and quality.

#### A framework for learning operators in chimp social hierarchies

**Figure 3:**
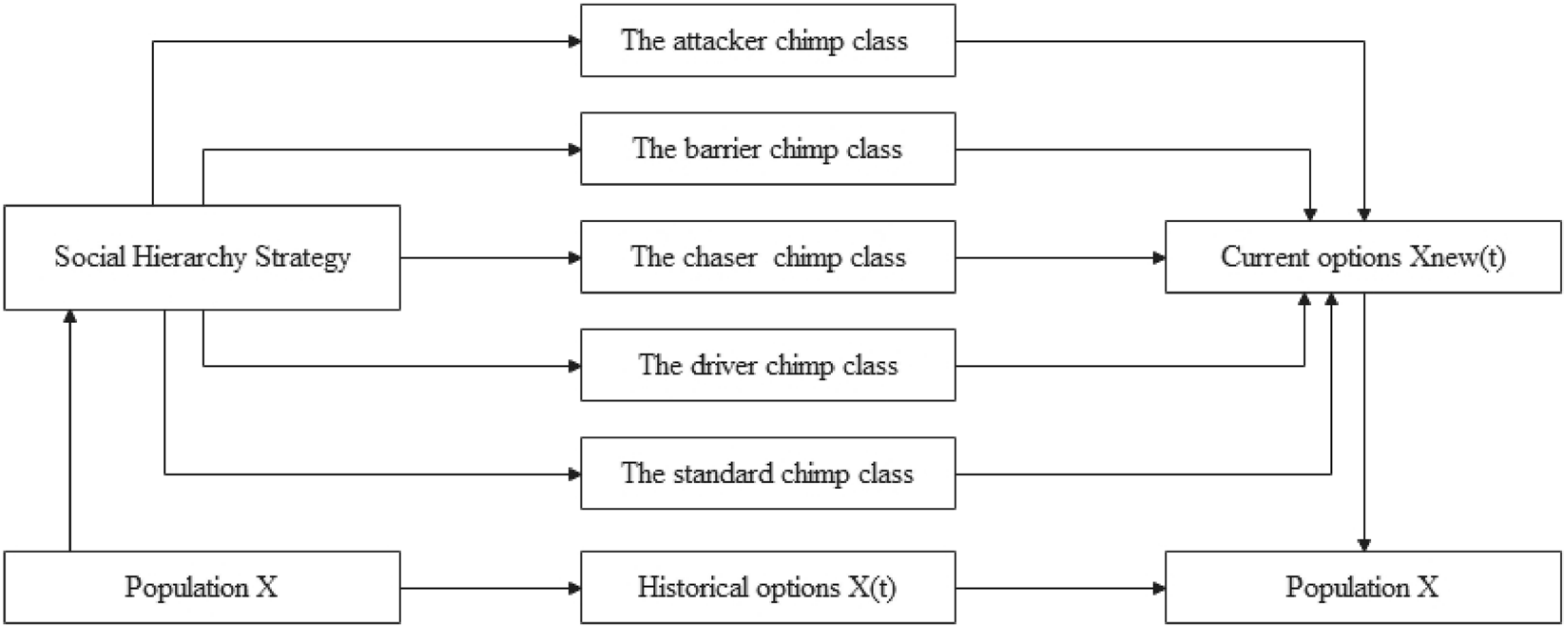
Framework of the CHoASH algorithm.

As can be seen in Fig. 3, the CHoASH operator framework is a straightforward structure which consists of the following two main parts:

- **Chimp social stratification.** Let the search space of the chimp population be a $N \times D$. *N* is the number of chimps, and *D* is the number of feature. The position of the *i* chimp at the time of *t* is ${X_i}\left( t \right) = \left( {x_{i,1}^t,x_{i,2}^t,x_{i,3}^t, \cdots ,x_{i,D}^t} \right)$ . In chimp social stratification, the population is divided into five social classes: the attacker chimp class, the barrier chimp class, the chaser chimp class, the driver chimp class, and the standard chimp class. We use ${S_i}\left( t \right)$ to describe the social class of each chimp. For example, if a chimp belongs to the attacker class, ${S_i}\left( t \right) = 1$. So, the barrier class, the chaser class, the driver class, and the standard chimp class are each ${S_i}\left( t \right) = 2$,${S_i}\left( t \right) = 3$,${S_i}\left( t \right) = 4$,${S_i}\left( t \right) = 5$. Then, the social hierarchy factor (SHF) is used to mark the hierarchical status of each chimp, which is calculated as
  17 $$\begin{aligned} SH{F_i}\left( t \right) = \frac{{L - {S_i}\left( t \right) }}{{L - 1}} \end{aligned}$$
  In Eq. (17), *L* represents the number of classes. Thus, if an individual chimp belongs to the attacker class, i.e., ${S_i}\left( t \right) = 1$, then the social class factor $SH{F_i}\left( t \right) = 1$. Then, when ${S_i}\left( t \right) = 2,SH{F_i}\left( t \right) = 0.75$. when ${S_i}\left( t \right) = 3,SH{F_i}\left( t \right) = 0.5$. when ${S_i}\left( t \right) = 4,SH{F_i}\left( t \right) = 0.25$. when ${S_i}\left( t \right) = 5,SH{F_i}\left( t \right) = 0$ .
- **Learning Strategies.** In the CHoASH algorithmic framework, two learning strategies are designed for different social classes: the attacking prey strategy and the autonomous search strategy. In the attacking prey strategy, individual chimps use the location information of chimps higher than their class to guide themselves to the region of the optimal solution. This strategy helps individual chimps to approach the optimal solution faster. In the autonomous search strategy, conversely, individual chimps observe information about the positions of chimpanzees higher than their rank and their position and update their position based on this information. This strategy allows chimp individuals to obtain more helpful information from higher-ranked individuals and thus improve their search behaviour. With the above two learning strategies, the CHoASH algorithm can consider local exploitation and global exploration, effectively improving the algorithm’s performance.

Therefore, when $SH{F_i}\left( t \right) > r$ is used at each iteration, the i-th chimp adopts the prey attack strategy at time *t*. Otherwise, it assumes the autonomous search strategy. Where the random number of $r \in \left[ {0,1} \right]$ . In the attacker stratum, $SH{F_i}\left( t \right) = 1$, and *r* is constantly less than or equal to 1, so individual chimp in this stratum have only the attack-prey strategy. In the common chimp class, $SH{F_i}\left( t \right) = 0$, and *r* is constantly greater than or equal to 0, so individual chimps in that class have only autonomous search strategies.

In the attacker chimp class, the position update equation is (18):

18 $$\begin{aligned} Xnew_{_{i,d}}^t = \left\{ {\begin{array}{*{20}{c}} {x_{A,k}^t\begin{array}{*{20}{c}} {\begin{array}{*{20}{c}} {\begin{array}{*{20}{c}} {\begin{array}{*{20}{c}} {\begin{array}{*{20}{c}} {}&{}{}&{}{} \end{array}}&{}{}&{}{} \end{array}}&{}{}&{}{} \end{array}}&{}{} \end{array}}&{}{}&{}{d \ne k} \end{array}}\\ {x_{A,d}^t + 2 \times f \times {r_1} \times \left( {x_{p,d}^t - x_{p,d}^t} \right) \begin{array}{*{20}{c}} {}&{}{d = k} \end{array}} \end{array}} \right. \end{aligned}$$

In Eq. (18), *d*, *k* is a random number in the interval $\left[ {1,D} \right]$ , *i*, *p*, *q* is a random number in the interval $\left[ {1,N} \right]$, and $i \ne p \ne q$ . $r_1$ is a random number in $\left[ {0,1} \right]$ .

In the barrier chimp class, the position update equation is (19):

19 $$\begin{aligned} Xnew_{_{i,d}}^t = \left\{ {\begin{array}{*{20}{c}} {x_{1,d}^t\begin{array}{*{20}{c}} {\begin{array}{*{20}{c}} {}&{}{}&{}{} \end{array}}&{}{}&{}{}&{}{{r_0} \ge 0.75} \end{array}}\\ {{{\left( {x_{A,d}^t + x_{B,d}^t} \right) } / 2}\begin{array}{*{20}{c}} {}&{}{}&{}{{r_0} < 0.75} \end{array}} \end{array}} \right. \end{aligned}$$

In the chaser chimp class, the position update equation is (20):

20 $$\begin{aligned} Xnew_{_{i,d}}^t = \left\{ {\begin{array}{*{20}{c}} {{{\left( {x_{1,d}^t + x_{2,d}^t} \right) } / 2}\begin{array}{*{20}{c}} {\begin{array}{*{20}{c}} {}&{}{}&{}{} \end{array}}&{}{}&{}{}&{}{{r_0} \ge 0.5} \end{array}}\\ {{{\left( {x_{A,d}^t + x_{B,d}^t + x_{C,d}^t} \right) } / 3}\begin{array}{*{20}{c}} {}&{}{}&{}{{r_0} < 0.5} \end{array}} \end{array}} \right. \end{aligned}$$

In the driver chimp class, the position update equation is (21):

21 $$\begin{aligned} Xnew_{_{i,d}}^t = \left\{ {\begin{array}{*{20}{c}} {{{\left( {x_{1,d}^t + x_{2,d}^t + x_{3,d}^t} \right) } / 3}\begin{array}{*{20}{c}} {\begin{array}{*{20}{c}} {}&{}{}&{}{} \end{array}}&{}{}&{}{}&{}{{r_0} \ge 0.25} \end{array}}\\ {{{\left( {x_{A,d}^t + x_{B,d}^t + x_{C,d}^t + x_{D,d}^t} \right) } / 4}\begin{array}{*{20}{c}} {}&{}{}&{}{{r_0} < 0.25} \end{array}} \end{array}} \right. \end{aligned}$$

In the standard chimp class, the position update equation is (22):

22 $$\begin{aligned} Xnew_{_{i,d}}^t = {{\left( {x_{1,d}^t + x_{2,d}^t + x_{3,d}^t + x_{4,d}^t} \right) } / 4} \end{aligned}$$

Note that a better solution may not be obtained through the learning strategy in Eqs. (18) to (22). Therefore, a screening mechanism is designed as follows:

23 $$\begin{aligned} x_{i,d}^{t + 1} = \left\{ {\begin{array}{*{20}{c}} {Xnew_{_{i,d}}^t,if\mathrm{{ }}f\left( {Xnew_{_{i,d}}^t} \right) < f\left( {x_{_{i,d}}^t} \right) }\\ {x_{_{i,d}}^t,otherwise} \end{array}} \right. \end{aligned}$$

From E. (23), the better of the current iteration chimp individual $Xnew_{_{i,d}}^t$ and the candidate chimp individual $x_{_{i,d}}^t$ will enter the next generation population.

In summary, the pseudo-code of CHoASH is shown in Algorithm 1.

**Algorithm 1:**
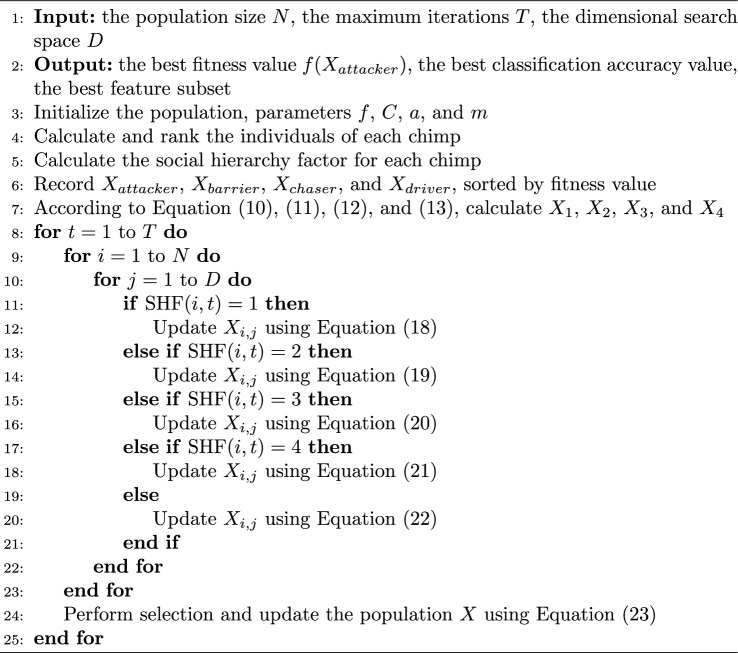
CHoASH Algorithm.

### Adaptive lens imaging oppositional learning strategies

During the iterative search process, ordinary chimp individuals in the chimp population are susceptible to being guided by attacker, barrier, chaser and driver as they gradually approach the optimal region. However, as the algorithm searches, all individuals in the chimpanzee population eventually converge on a narrow area. This situation may cause the algorithm to fall into a local optimum, especially when the attacker is a local optimum, and the CHoA algorithm is prone to fall into a local optimum.

**Figure 4:**
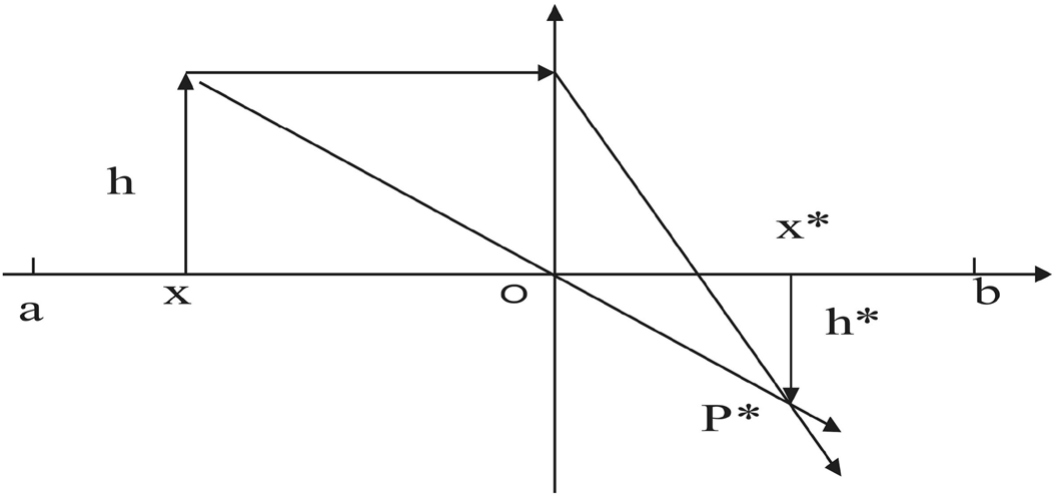
Lens imaging oppositional learning strategy.

To enhance the global exploration capability of the CHoA algorithm and make it jump out of the local optimum, we introduce an adaptive oppositional learning strategy based on the lens imaging principle. The main idea of this strategy is to generate new individuals by observing the behavioural patterns of the current optimal individual and analyzing them inversely using the lens imaging principle. Now, let the feasible solution *X* in the solution space; there always exists a corresponding inverse solution ${X^*}$. Suppose the new individual solution ${X^*}$ is better than the solution *X* of the current optimal individual. In that case, it makes the algorithm more exploratory and thus avoids the plague of local optimal solutions. The advantage of this strategy is that these new individuals are added to the algorithm to compete and evolve with the current population to find better solutions. Figure 4 shows the one-dimensional optimal individual (*x* ) space learning process based on the lens imaging principle.

In Fig. 4, there is an individual *P* with height *h*; its projection on the coordinate axis is *x* ( *x* is the global optimal individual). The base position is *o* (in this paper, we take the midpoint of $\left[ {a,b} \right]$ ) on the placement of the lens with focal length *f*, and through the process of lens imaging to obtain a height of ${h^*}$ image ${P^*}$, its projection on the coordinate axis is ${x^*}$. Therefore, the global optimal individual *x*, obtained based on the lens imaging oppositional learning strategy, produces the inverse individual as ${x^*}$. The following equation can be derived based on the principle of convex lens imaging in Fig. 3 and the oppositional learning strategy of lens imaging in Fig. 4.

24 $$\begin{aligned} \frac{{\frac{{a + b}}{2} - x}}{{{x^*} - \frac{{a + b}}{2}}} = \frac{h}{{{h^*}}} \end{aligned}$$

Now let $\frac{h}{{{h^*}}} = g$ , the transformation of Eq. (24) to solve the inverse solution ${x^*}$ is given below:

25 $$\begin{aligned} {x^*} = \frac{{a + b}}{2} + \frac{{a + b}}{{2g}} - \frac{x}{g} \end{aligned}$$

From Eq. (25), assuming that the base point *o* is fixed, the larger the regulator *g* is, the closer the inverse solution is to the base point *o* and the closer it is to the feasible solution. Therefore, the regulating factor, called the micro-regulator, searches only a small area around the possible solution, increasing the population’s diversity. In general, generalizing the oppositional learning strategy based on the convex lens imaging principle shown in (26) to the *D* dimensional space yields:

26 $$\begin{aligned} x_d^* = \frac{{{a_d} + {b_d}}}{2} + \frac{{{a_d} + {b_d}}}{{2g}} - \frac{x}{g} \end{aligned}$$

Where ${x_d}$ and $x_d^*$ are the d-th dimension components of *x* and ${x^*}$, respectively, ${a_d}$ and${b_d}$ are the *d* dimension components of the upper and lower bounds of the decision variables, respectively. Meanwhile, it can also be seen from Eq. (26) that the modulation factor *g* is an important parameter that affects the learning performance of lens imaging. Considering that a smaller value of *g* generates a more extensive range of inverse solutions, while a more significant deal of *g* causes a small range of inverse solutions, combined with the characteristics of the CHoA algorithm’s large-scale exploration in the pre-iterative stage and the local refined search in the post-iterative location, this paper proposes a kind of adaptive regulating factor that varies with the number of iterations:

27 $$\begin{aligned} g = {\left( {1 + {{\left( {\frac{t}{T}} \right) }^{0.5}}} \right) ^{10}} \end{aligned}$$

*t* is the current iteration number, and *T* is the maximum iteration number. Since *g* in Eq. (27) is used as the denominator to regulate the inverse solution, the value of *g* becomes larger as the number of iterations increases. The range of the inverse solution of the lens imaging oppositional learning becomes smaller and smaller. This regulation enlarges the ability of the algorithm to develop globally at the later stage of iteration and improves the diversity of the population.

The opposing solution generated by adaptive lens imaging oppositional learning is not necessarily superior to the original solution. Therefore, a screening mechanism is introduced to select whether to replace the original solution with the inverse solution, i.e., only if the inverse solution has a better fitness value. The formula is as follows:

28 $$\begin{aligned} {x_{i,d}} = \left\{ {\begin{array}{*{20}{c}} {x_{_{i,d}}^*,if\mathrm{{ }}f\left( {x_{_{i,d}}^*} \right) < f\left( {{x_{i,d}}} \right) }\\ {{x_{i,d}},otherwise} \end{array}} \right. \end{aligned}$$

Algorithm 2, which provides an adaptive lens imaging strategy for the specific steps, are as follows:

**Algorithm 2:**
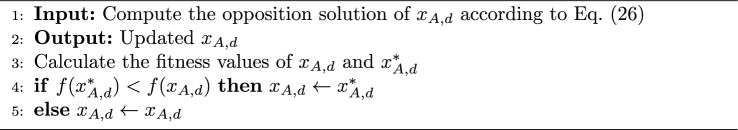
Adaptive Lens Imaging Oppositional Learning.

### Binary ALI-CHoASH

To solve the feature selection problem, this paper binaries the improved algorithm ALI-CHoASH. In the binaryised ALI-CHoASH, all the solutions in the solution space are converted to binary form with the value range of [0,1]. The conversion function for converting solutions from continuous values to binary format is shown in Eq. (29).

29 $$\begin{aligned} x_i^j = \left\{ {\begin{array}{*{20}{c}} {0,f\left( {x_i^j} \right) < 0.5}\\ {1,f\left( {x_i^j} \right) > 0.5} \end{array}} \right. \end{aligned}$$

Where the individual *i* has a fitness value of $f\left( {x_i^j} \right)$.

The feature subsets selected by the ALI-CHoASH algorithm are all evaluated by the KNN classifier. Since the feature selection problem aims to find the smallest subset of features with maximum classification accuracy, our fitness function is set to the form shown in Eq. (30).

30 $$\begin{aligned} f\left( {{X_i}} \right) = \alpha \cdot Err + \left( {1 - \alpha } \right) \times \left( {\frac{{\left| R \right| }}{{\left| C \right| }}} \right) \end{aligned}$$

*Err* denotes the classification error rate, $\left| R \right|$ denotes the number of selected feature sets, $\left| C \right|$ denotes the number of original feature sets, and $\alpha$ denotes the weighting factor. Since Eq. (30) plays a massive role in searching the optimal subset of features for the ALI-CHoASH algorithm, $\alpha$ is set to 0.99.

**Figure 5:**
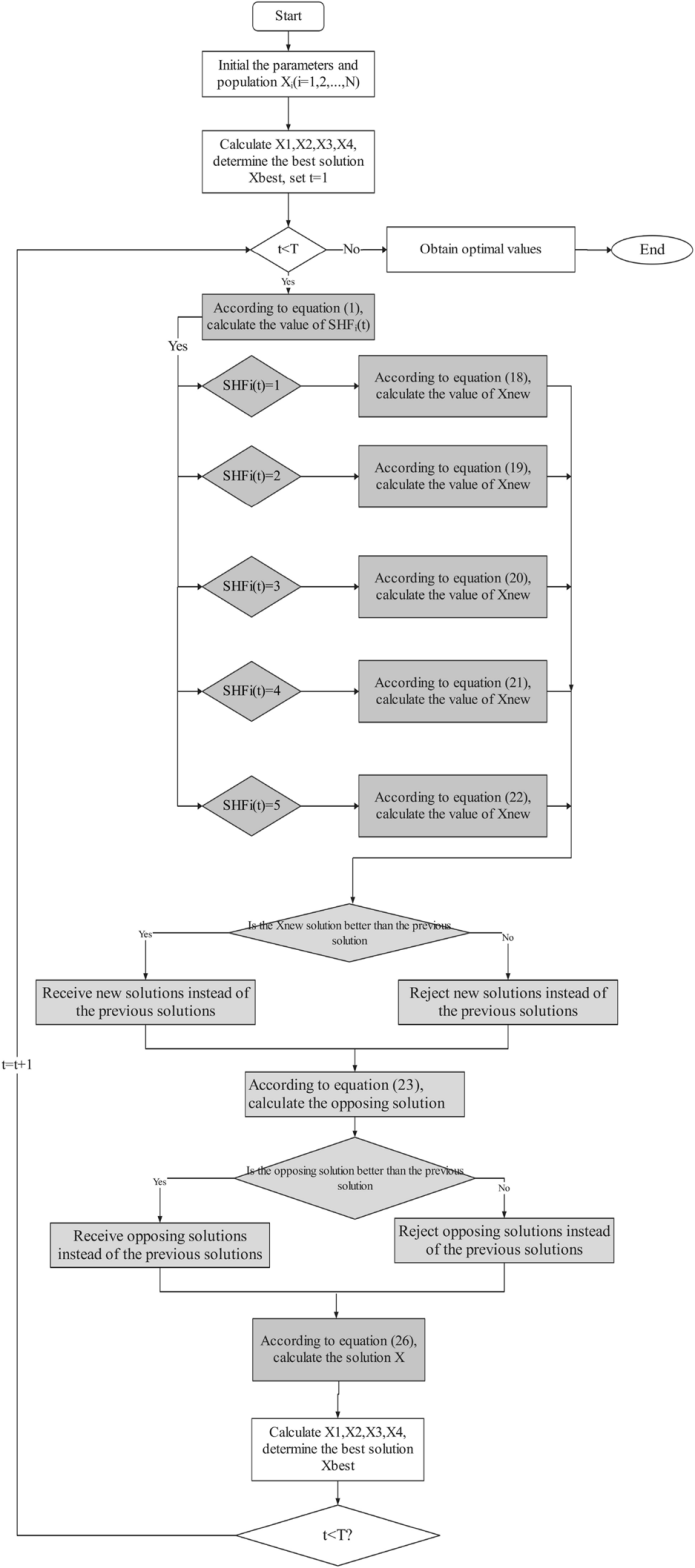
The flow chart of the ALI-CHoASH algorithm.

In summary, the flowchart of the ALI-CHoASH method is shown in Fig. 5.

## Experimental analyses and discussions

To evaluate the comprehensive performance of ALI-CHoASH. This section conducts a series of comparative experiments to validate it, and the detailed description of the adopted categorical dataset is shown in Table 1. Firstly, the setup of the comparison algorithms is described; secondly, the level of exploration and exploitation in the ALI-CHoASH algorithm is measured and quantitatively analyzed in terms of diversity, and the search strategies affecting these two factors are practically analyzed. Thirdly, the relationship between the classification performance and the number of features in the ALI-CHoASH algorithm is investigated; fourthly, multifaceted performance assessments such as classification accuracy, dimensionality approximation, convergence and stability are performed. Finally, the comparison algorithms’ convergence performance and Wilcoxon rank sum test are verified. Python was used as the programming language in the experiments. All the experiments were executed on a Legion machine with Inter Core i5 CPU (3.20GHz), and 8G RAM, and all the algorithms were tested using Pycharm2021.

### Datasets

Six UCI (https://archive.ics.uci.edu/), six ASU (https://jundongl.github.io/scikit-feature/datasets.html) and four gene (https://ckzixf.github.io/dataset.html) datasets from the database to verify the performance of ALI-CHoASH.During the experiment, for each dataset in Table 1, 70% of the samples were randomly selected as training data and 30% as test data. In addition, the experiments were conducted using a KNN classifier to evaluate each of the obtained feature subsets. Table 1 briefly describes these datasets, with samples ranging from 60 to 1560, features ranging from 14 to 11225, and class labels ranging from 2 to 26. When the number of class labels is two categories, it is considered binary. When the number of class labels is more significant than two classes, it is considered multicategory.

**Table 1:** Test data set.

No.	Data set	#Instances	#Features	#Classes	Data sources
1	Wine	177	14	3	UCI
2	HeartEW	269	14	2	UCI
3	Zoo	100	17	7	UCI
4	Vote	299	17	2	UCI
5	Congress	434	17	2	UCI
6	BreastEW	568	31	2	UCI
7	lung_discrete	73	325	7	ASU
8	Isolet	1560	617	26	ASU
9	colon	62	2000	2	ASU
10	lung	203	3312	5	ASU
11	Leukemia_1	72	5327	3	ASU
12	DLBCL	77	5469	2	Gene
13	9_Tumor	60	5726	9	Gene
14	leukemia	72	7070	2	ASU
15	Leukemia_2	72	7129	4	Gene
16	Leukemia_3	72	11225	3	Gene

### Algorithm parameterization and evaluation metrics

To ensure the fairness of the result comparison, all the experiments in this paper are conducted in the same environment. For each test dataset, the experiments are executed *M* times (its value is set to 30 times) to evaluate the feature selection performance of each algorithm. *T* is the maximum number of iterations of the algorithm run (its value is 100 times), and *t* denotes the number of current iterations. To reduce the computational cost and maintain the search efficiency, the number of populations is uniformly set to 10. To verify the optimization effect of the proposed methods in the feature selection process, the exploration and exploitation percentage, average classification accuracy, average number of selected features, average optimal fitness value and optimal fitness value are used to evaluate the performance of the algorithms, as shown in Eqs. (33) to (39). In addition, a statistical significance test, i.e., the nonparametric Wilcoxon rank sum test, was performed, and the significance level in the statistical significance test was chosen to be 0.05. The pre-set parameters for each algorithm are shown in Table 2.

**Table 2:** Parameter setting of the comparison algorithm.

Name of algorithm	Description/year of publication	Parameter values
ALI-CHoASH	Adaptive Lens Imaging Chimp Social Hierarchy Algorithm	${r_1} \in \left[ {0,1} \right] ,{r_2} \in \left[ {0,1} \right] ,f \in \left( {0,2.5} \right) ,u \in \left[ {0,1} \right]$
CHoA	Chimp optimization algorithm/2020^21^	${r_1} \in \left[ {0,1} \right] ,{r_2} \in \left[ {0,1} \right] ,f \in \left( {0,2.5} \right) ,u \in \left[ {0,1} \right]$
SChoA	sine-cosine chimp optimization algorithm/2022^26^	${r_1} \in \left[ {0,1} \right] ,{r_2} \in \left[ {0,1} \right] ,f \in \left( {0,2.5} \right) ,u \in \left[ {0,1} \right]$
GWO	Grey Wolf Optimizer/2014^11^	${r_1} \in \left[ {0,1} \right] ,{r_2} \in \left[ {0,1} \right] ,a \in \left( {0,2.5} \right)$
SSA	Salp Swarm Algorithm/2017^12^	$m=2$
HHO	Harris hawks optimization(HHO)/2019^13^	$p = 0.5,J \in \left[ {0,2} \right]$
SMA	Slime mould algorithm/2020^14^	$\delta = 0.03$
BES	Bald Eagle Search optimisation algorithm/2020^15^	$\alpha = 1.5,a = 10,R = 1,{c_1} = {c_2} = 2$
GMPBSA	backtracking search algorithm driven by generalized mean position/2023^29^	$DIM\_RATE\mathrm{{ }} = \mathrm{{ }}1$

**Figure 6:**
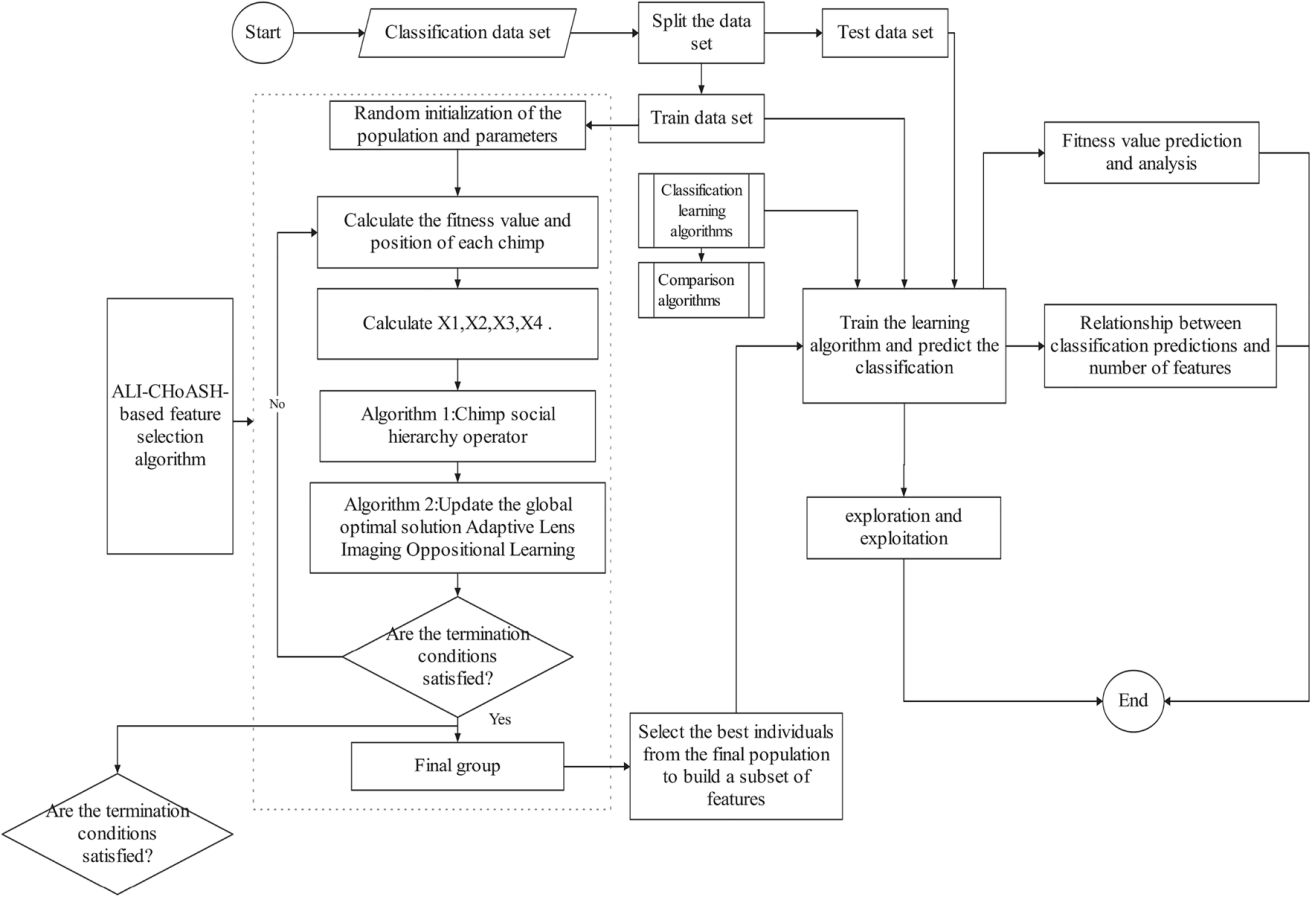
Experimental framework diagram.

To evaluate the effect of the ALI-CHoASH algorithm on data classification performance during feature selection, three sets of comparison experiments are designed as follows. In the first set of comparison experiments, ALI-CHoASH will be compared with the CHoA and SCHoA algorithms regarding exploration and exploitation percentage, average fitness value, optimal fitness value and classification performance. In the second set of experiments, the relationship between the classification performance of the ALI-CHoASH algorithm and the number of features will be investigated. In the third set of comparison experiments, ALI-CHoASH will be compared with GWO, SSA, HHO, SMA, BES and GMPBSA regarding fitness value and classification performance, respectively. The experimental framework is shown in Fig. 6. The specific technical routes of the experiments are as follows: firstly, ALI-CHoASH is run on the training dataset to generate a subset of candidate features and output the subset of features with the best performance; secondly, the training and test sets are converted into new training and testing set by removing the unselected features; then the classification algorithms are trained on the transformed training dataset; and finally, the converted test dataset into the learned classifier to verify the classification performance of the selected feature subset and the selected feature subset of the comparison algorithm.

Diversity refers to the degree of distribution of individuals in the solution space, which helps to ensure that the algorithm searches widely in the solution space and avoids locally optimal solutions. The following formula is used to measure diversity.

31 $$\begin{aligned} Di{v_j}= & {} \frac{1}{n}\sum \limits _{i = 1}^n {median\left( {{x^j}} \right) } - x_i^j \end{aligned}$$

32 $$\begin{aligned} Div= & {} \frac{1}{D}\sum \limits _{j = 1}^D {Di{v_j}} \end{aligned}$$

$median\left( {{x^j}} \right)$ represents the median of dimension *j* in the whole population, and *Div* represents the diversity of the entire population during the iteration process. $Di{v_j}$ represents the diversity of all individuals in dimension *j*.

Percentage of exploration: Indicates the percentage of investigation per iteration in the algorithm, calculated as follows.

33 $$\begin{aligned} Xpl\% = \frac{{Div}}{{Di{v_{\max }}}} \times 100 \end{aligned}$$

Development Percentage: Indicates the percentage of development per iteration in the algorithm, calculated as follows.

34 $$\begin{aligned} Xpt\% = \frac{{\left| {Div - Di{v_{\max }}} \right| }}{{Di{v_{\max }}}} \times 100 \end{aligned}$$

Where *Div* is the diversity of the cluster in the iteration and $Di{v_{\max }}$ is the maximum diversity in all iterations.

Average Classification Accuracy: represents the average of the classification accuracy of the selected feature set, where $acc\left( i \right)$ is the accuracy of the i-th classification, calculated as follows.

35 $$\begin{aligned} AccMean = \frac{1}{M}\sum \limits _{i = 1}^M {acc\left( i \right) } \end{aligned}$$

Average number of selected features: describes the average of the classification accuracy of the selected set of features, where $number\left( i \right)$ is the number of features selected for the ith time, which is calculated as follows.

36 $$\begin{aligned} AccMean = \frac{1}{M}\sum \limits _{i = 1}^M {acc\left( i \right) } \end{aligned}$$

Average fitness value: the average of the mean fitness values of the resulting solutions is calculated, where $fitness\left( i \right)$ is the i-th fitness value, which is calculated as follows.

37 $$\begin{aligned} FitMean = \frac{1}{M}\sum \limits _{i = 1}^M {fitness\left( i \right) } \end{aligned}$$

Average optimal fitness value: Calculate the minimum fitness values. This is calculated as follows.

38 $$\begin{aligned} \min Fit = \min \left\{ {fitness\left( 1 \right) ,fitness\left( 2 \right) , \cdots ,fitness\left( i \right) } \right\} \end{aligned}$$

Average Running Time: The average running time of the classification method for each dataset, where $Runtime\left( i \right)$ is the time consumed in the i-th run, is calculated as follows.

39 $$\begin{aligned} TimeMean = \frac{1}{M}\sum \limits _{i = 1}^M {Runtime\left( i \right) } \end{aligned}$$

### Results and discussion

#### ALI-CHoASH and CHoA diversity analysis

**Figure 7:**
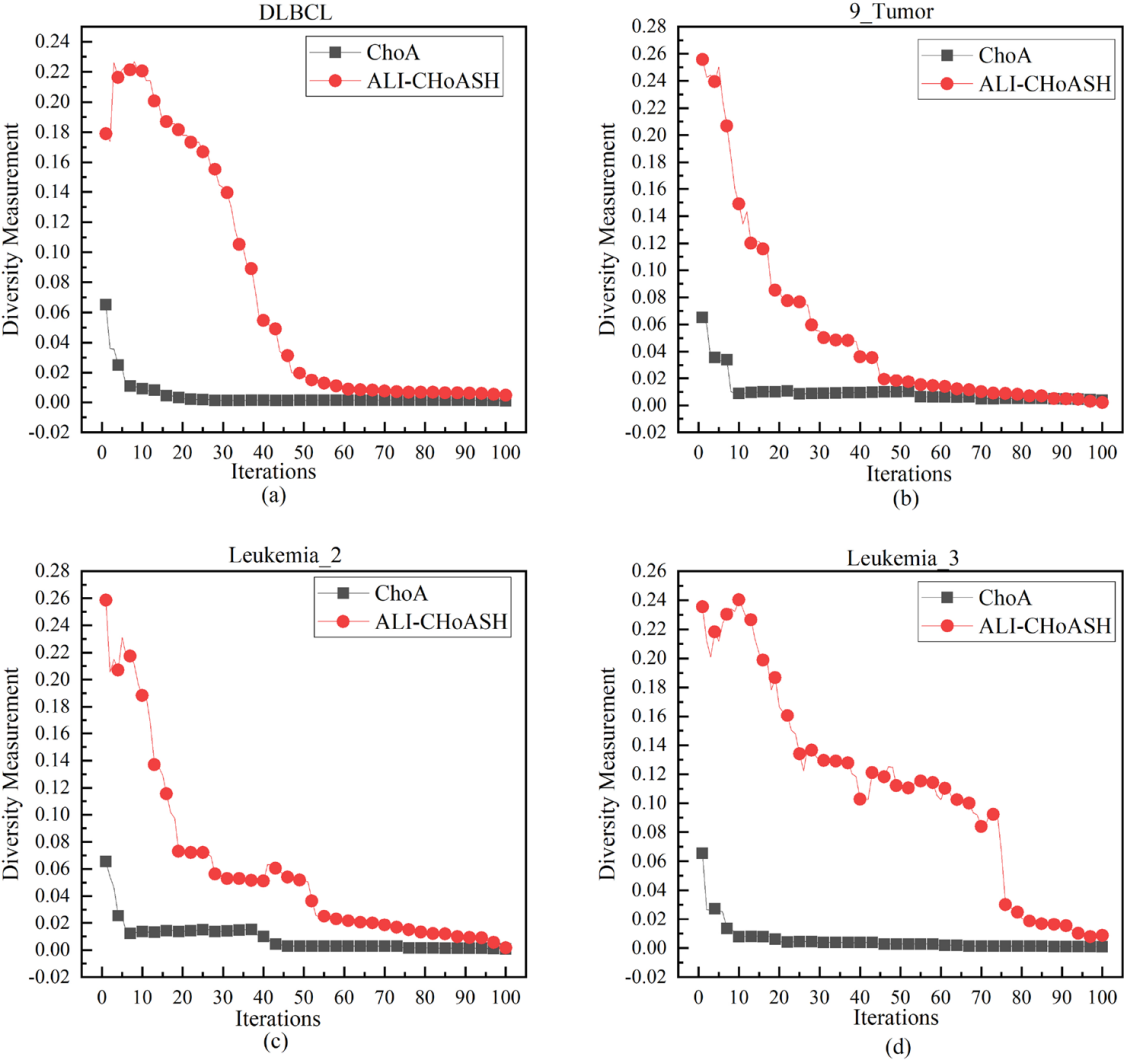
ALI-CHoASH and CHoA diversity in the gene dataset.

**Figure 8:**
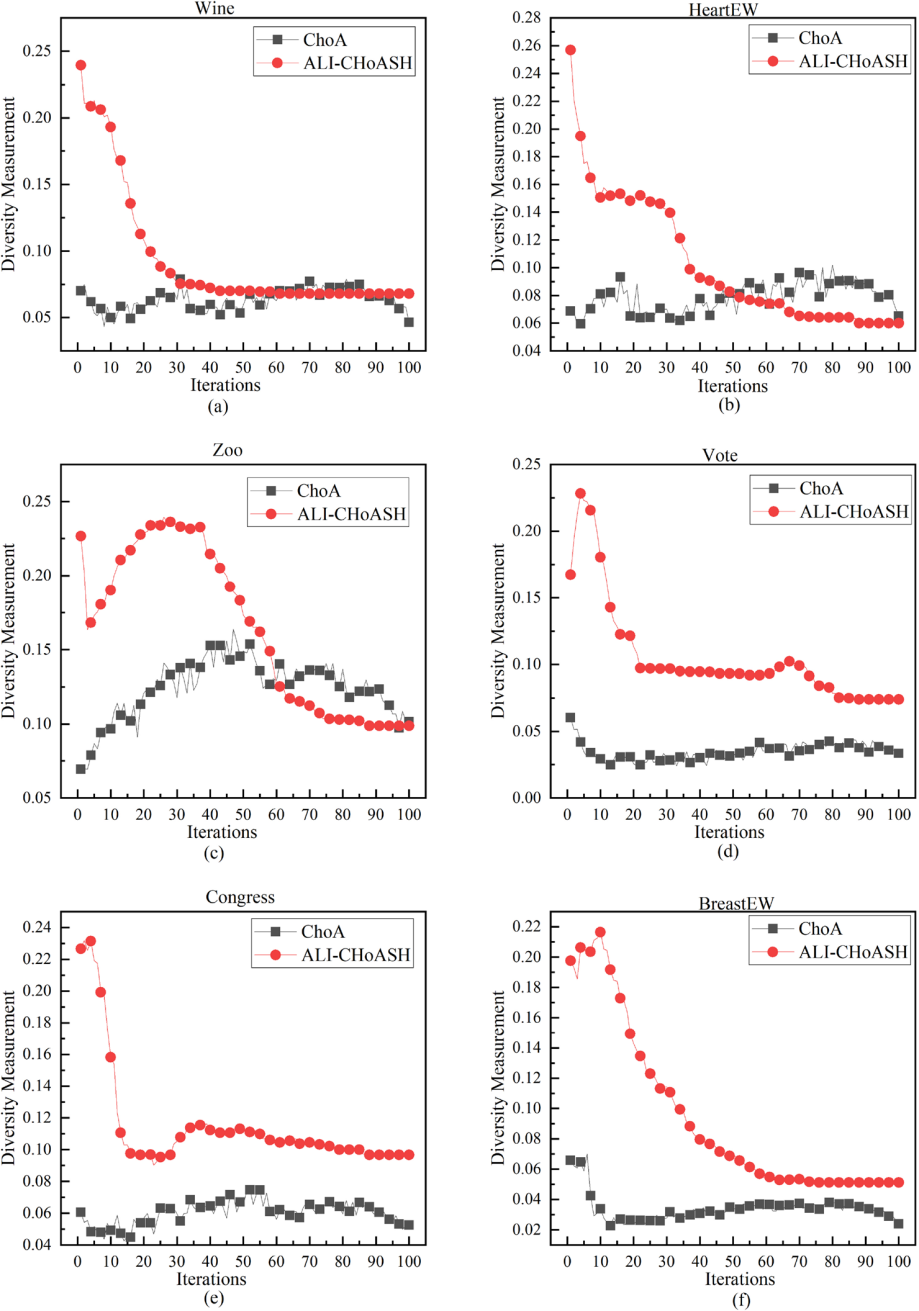
ALI-CHoASH and CHoA diversity in the UCI dataset.

**Figure 9:**
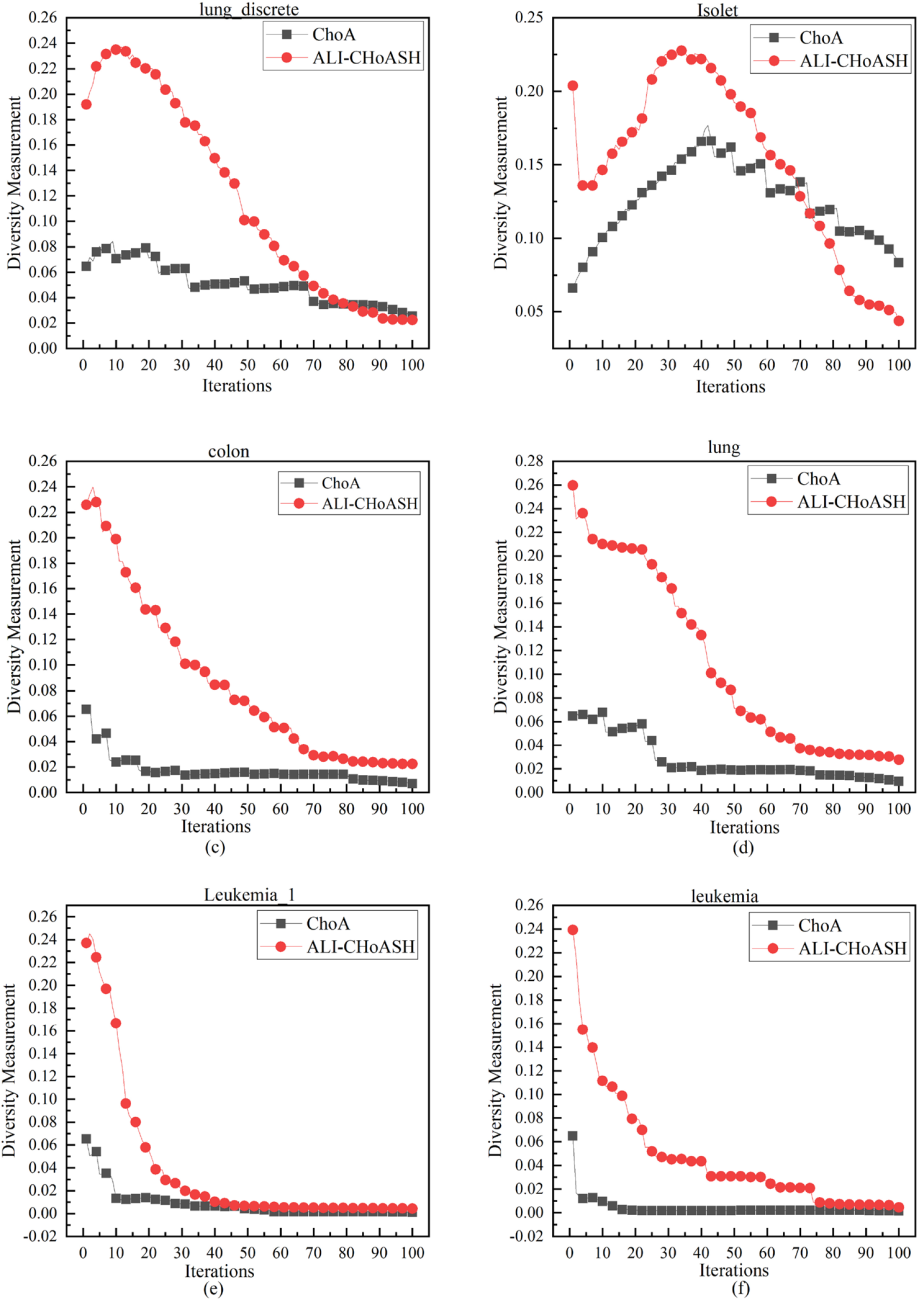
ALI-CHoASH and CHoA diversity in the ASU dataset.

Maintaining diversity in algorithms has several benefits. These include increasing the search space, improving algorithm performance and robustness, and avoiding premature convergence. The measured diversity of the ALI-CHoASH and CHoA algorithms during the iteration period is shown in Figs. 7, 8 and 9. The experiments on 16 datasets demonstrate that the ALI-CHoASH algorithm has a more robust diversity than the CHoA algorithm. The ALI-CHoASH algorithm enhances individual interaction and communication, accelerates information dissemination, and improves group collaboration efficiency and effectiveness. Moreover, the algorithm helps the group eliminate local optimal solutions and search for global ones.

#### Discussion of the results of the ALI-CHoASH with CHoA and SCHoA experiments

Table 3 shows the optimal fitness values and feature subsets for different algorithms. Table 3 shows that ALI-CHoASH achieves better optimal fitness values on all test datasets than CHoA and SCHoA. And on Vote, Congress, lung_discrete, Isolet, Leukemia_1 and Leukemia_3 datasets, ALI-CHoASH selects the minimum number of feature subsets.

**Table 3:** Number of feature selections and Optimal fitness values for ALI-CHoASH and its enhanced algorithms.

Data name	Optimal fitness values			Number of feature selections		
	ALI-CHoASH	CHoA	SCHoA	ALI-CHoASH	CHoA	SCHoA
Wine	**2.97E−03**	5.05E−02	4.39E−02	3.07	2.9	3.03
HeartEW	**1.04E−01**	1.70E−01	1.41E−01	3.33	1.73	2.57
Zoo	**1.98E−02**	6.90E−02	6.61E−02	5.3	4.8	5.37
Vote	**1.13E−02**	1.73E−02	1.54E−02	2.8	3.1	3.47
Congress	**6.92E−03**	2.98E−02	2.54E−02	1.4	1.27	1.97
BreastEW	**4.13E−02**	5.71E−02	5.56E−02	4.1	2.13	2.33
lung_discrete	**3.03E−02**	8.14E−02	4.26E−02	11.03	11.4	18.67
Isolet	**9.30E−02**	1.25E−01	1.28E−01	179.8	189.6	229.27
Colon	**5.80E−05**	9.55E−02	5.73E−02	11.6	3.9	6.3
Lung	**6.61E−03**	2.34E−02	1.80E−02	40.1	46.4	37.93
Leukemia_1	**3.02E−03**	3.75E−02	2.41E−02	13.23	25.87	65.73
DLBCL	**1.13E−05**	3.71E−02	2.06E−02	6.2	3.5	5.6
9_Tumor	**3.39E−01**	4.40E−01	4.24E−01	62.23	14.73	20.97
Leukemia	**9.05E−06**	3.60E−02	1.50E−02	6.4	2.7	8.9
Leukemia_2	**1.81E−02**	9.90E−02	7.35E−02	98.63	9.6	15.1
Leukemia_3	**1.33E−05**	3.07E−05	3.61E−05	14.97	34.43	40.5

Best value in each row of the table is identified in bold.

**Figure 10:**
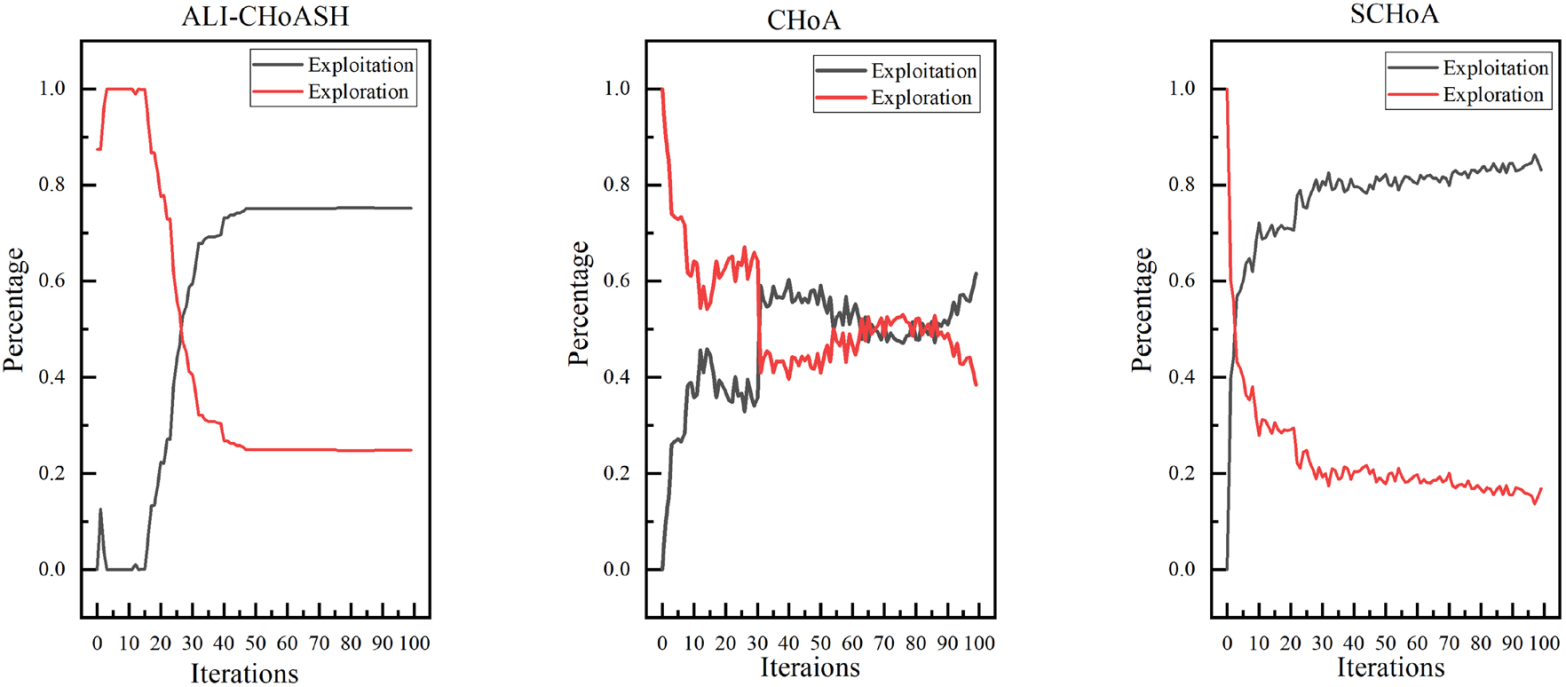
Average exploration and exploitation of Wine.

**Figure 11:**
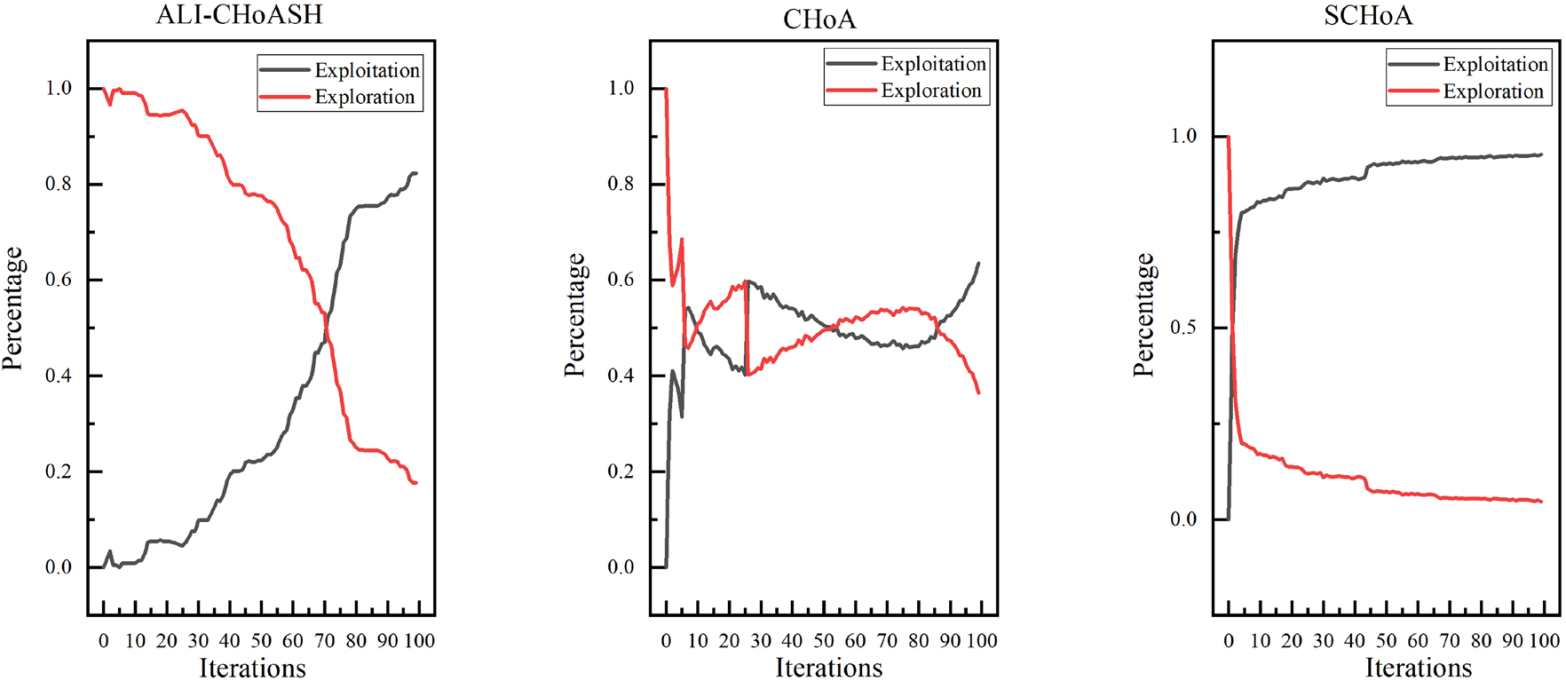
Average exploration and exploitation of lung_discrete.

**Figure 12:**
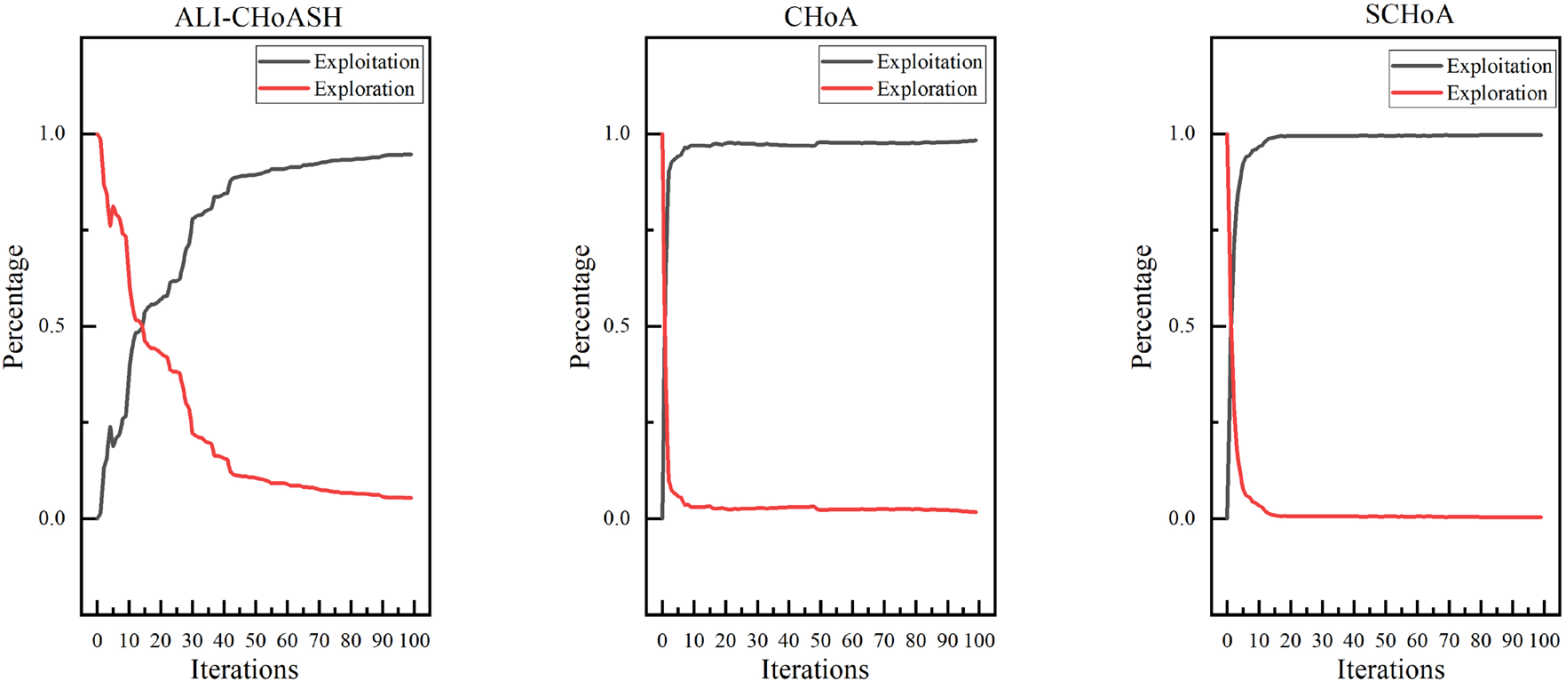
Average exploration and exploitation of colon.

**Figure 13:**
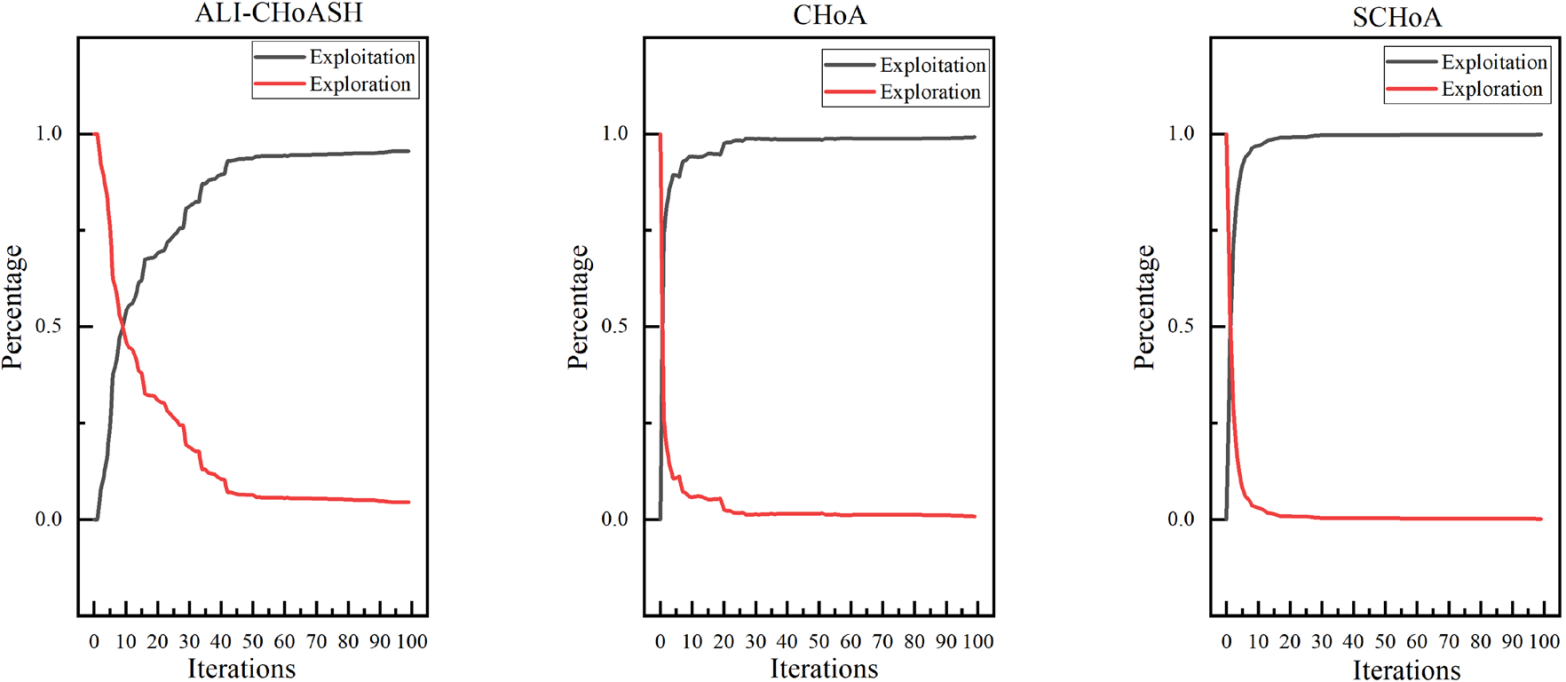
Average exploration and exploitation of leukemia.

Exploration and exploitation capabilities have a significant impact on optimization performance. Existing meta-heuristic algorithm analyses only compare the final version of classifications^40,41^ but cannot assess the balance between exploration and exploitation. Therefore, experimental studies based on diversity measurements are needed to evaluate the exploration and exploitation capabilities of ALI-CHoASH quantitatively. As seen from Table 4, ALI-CHoASH achieves better average fitness values on all test datasets than CHoA and SCHoA. Also, the percentage of exploration and exploitation completed by ALI-CHoASH is relatively more balanced on all test datasets. For example, as seen from the Wine dataset in Table 4, the percentage of exploration and exploitation achieved by ALI-CHoASH is 55.73%:44.27%. It can be observed from Fig. 10 that in the first about ten iterations, ALI-CHoASH shows a clear tendency to enhance the exploration search space. After that, the ALI-CHoASH algorithm significantly improves and maintains a clear direction to expand the exploration space. This phenomenon shows that the algorithm introduces a social class multiple learning strategies and an adaptive lens imaging oppositional learning strategy, which prolongs the exploration effect and prevents a sharp decline in population diversity. Such optimization strategies give the algorithm a more robust global search and local convergence performance and high efficiency and accuracy in solving complex optimization problems. The percentage of exploration and exploitation achieved by CHoA is 76.09%:23.91%. In the first about 30 iterations, CHoA shows a clear tendency to enhance the exploration search space. After that, the CHoA algorithm’s exploitation capability is significantly improved. The exploration and exploitation capabilities alternately appear to be enhanced during the subsequent iterations, which results in a sharp decrease in population diversity. The lung_discrete dataset in Table 4 shows that the percentage of exploration and exploitation achieved by ALI-CHoASH is 67.38%:32.62%. It can be observed from Fig. 11 that in the first about 70 iterations, ALI-CHoASH shows a clear tendency to expand the exploration search space. After that, the ALI-CHoASH algorithm’s exploitation capability significantly improves and maintains a clear direction to expand the exploration space. Such a result is favourable to preventing a sharp decline in population diversity. While the percentage of exploration and exploitation achieved by CHoA is 76.09%:23.91%. The rate of exploration and exploitation completed by SCHoA is 11.00%:89.00%. For the first approximately 70 iterations, SCHoA shows a clear tendency to explore the search space. After that, the SCHoA algorithm’s ability to exploit was significantly improved. The exploration and exploitation capabilities then maintain an equilibrium state during the subsequent iterations, which results in a sharp decrease in population diversity. The colon dataset in Fig. 12 and Table 4 shows that the percentage of exploration and exploitation achieved by ALI-CHoASH is 23.85%:76.15%. In contrast, the percentage of exploration and exploitation conducted by CHoA is 4.08%:95.92%. The rate of exploration and exploitation completed by SCHoA is 2.96%:97.04%. The leukemia dataset in Fig. 13 and Table 4 shows that the percentage of exploration and exploitation achieved by ALI-CHoASH is 18.99%:81.01%. In contrast, the rate of exploration and exploitation completed by CHoA is 3.73%:96.27%. The portion of exploration and exploitation conducted by SCHoA is 2.86%:97.14%. Combining the above descriptions, it is clear that when the percentages of exploration and exploitation are relatively balanced, it is possible to prevent a sharp decline in population diversity, thus contributing to an increase in the fitness value.

**Table 4:** Average Xpl%:Xpt% and Average fitness values for ALI-CHoASH and its enhanced algorithms.

Data name	Average Exploration%:Exploitation			Average fitness values		
	ALI-CHoASH	CHoA	SCHoA	ALI-CHoASH	CHoA	SCHoA
Wine	55.73%:44.27%	76.09%:23.91%	45.33%:54.68%	**8.65E−03**	5.66E−02	4.57E−02
HeartEW	45.00%:55.00%	68.70%:31.30%	37.55%:62.45%	**1.11E−01**	1.72E−01	1.43E−01
Zoo	68.64%:31.36%	26.59%:73.41%	46.36%:53.65%	**3.11E−02**	7.44E−02	6.79E−02
Vote	38.73%:61.27%	60.95%:39.05%	42.16%:57.84%	**1.35E−02**	1.78E−02	1.55E−02
Congress	69.43%:30.57%	24.96%:75.04%	35.58%:64.42%	**7.66E−03**	2.98E−02	2.57E−02
BreastEW	43.55%:56.33%	52.90%:47.10%	22.90%:77.10%	**4.39E−02**	5.83E−02	5.59E−02
lung_discrete	67.38%:32.62%	50.88%:49.12%	11.00%:89.00%	**5.51E−02**	8.34E−02	4.38E−02
Isolet	67.05%:32.95%	88.82%:11.18%	63.17%:36.83%	**1.07E−01**	1.27E−01	1.29E−01
Colon	23.85%:76.15%	4.08%:95.92%	2.96%:97.04%	**1.43E−02**	1.05E−01	6.48E−02
Lung	24.87%:75.13%	21.59%:78.41%	6.82%:93.18%	**1.15E−02**	2.50E−02	1.87E−02
Leukemia_1	20.21%:79.79%	25.99%:74.01%	8.17%:91.84%	**5.90E−03**	4.08E−02	2.58E−02
DLBCL	29.73%:70.27%	17.23%:82.77%	4.33%:95.67%	**7.00E−03**	4.00E−02	2.35E−02
9_Tumor	20.75%:78.46%	16.86%:83.14%	6.75%:93.25%	**4.03E−01**	4.50E−01	4.33E−01
Leukemia	18.99%:81.01%	3.73%:96.27%	2.86%:97.14%	**8.83E−04**	3.95E−02	2.09E−02
Leukemia_2	47.88%:52.12%	8.64%:91.36%	7.16%:92.84%	**2.39E−02**	1.08E−01	7.95E−02
Leukemia_3	37.10%:62.90%	8.22%:91.78%	3.43%:96.57%	6.02E−03	1.62E−04	1.44E−04

Best value in each row of the table is identified in bold.

Table 5 shows the average accuracy and runtime of the different algorithms. ALI-CHoASH achieves higher average classification accuracy on all test datasets. Also, the runtime of the ALI-CHoASH algorithm is well within the acceptable range.

**Table 5:** The running time (/s) and classification accuracy of CHoA algorithms.

Data name	Classification accuracy			Time (/s)		
	ALI-CHoASH (%)	CHoA (%)	SCHoA (%)	ALI-CHoASH	CHoA	SCHoA
Wine	**99.94**	95.12	95.80	11.65	2.43	2.95
HeartEW	**89.79**	82.96	85.93	14.88	1.96	3.03
Zoo	**98.33**	93.33	93.67	8.52	2.66	2.84
Vote	**99.78**	98.44	98.67	18.45	3.39	3.88
Congress	**99.39**	97.07	97.56	22.02	3.16	4.47
BreastEW	**95.96**	94.31	94.46	27.99	5.65	6.61
lung_discrete	**96.97**	91.82	95.76	17.4	9.55	11.78
Isolet	**90.90**	87.69	87.40	165.6	49.08	52.77
Colon	**100.00**	90.35	94.21	65.41	44.34	57.29
Lung	**99.34**	97.65	98.20	63.3	80.18	104.85
Leukemia_1	**99.70**	96.21	97.58	178.52	122.11	163.52
DLBCL	**100.00**	96.25	97.92	165.78	121.25	162.3
9_Tumor	**65.74**	55.56	57.22	439.09	385.01	425.82
Leukemia	**100.00**	96.36	98.48	234.9	161.66	215.85
Leukemia_2	**98.18**	90.00	92.58	224.38	162.13	216.86
Leukemia_3	**100.00**	100.00	100.00	363.89	245.82	327.46

Best value in each row of the table is identified in bold.

In conclusion, ALI-CHoASH shows better performance than SCHoA and ChoA algorithms in terms of optimal fitness value, average fitness value, average classification accuracy, robustness, and percentage of exploration and exploitation, and proves that ALI-CHoASH’s ability to explore and exploit as well as its ability to jump out of the local optimum is somewhat superior.

#### Analyzing classification performance in ALI-CHoASH to the correlation between the number of features

**Figure 14:**
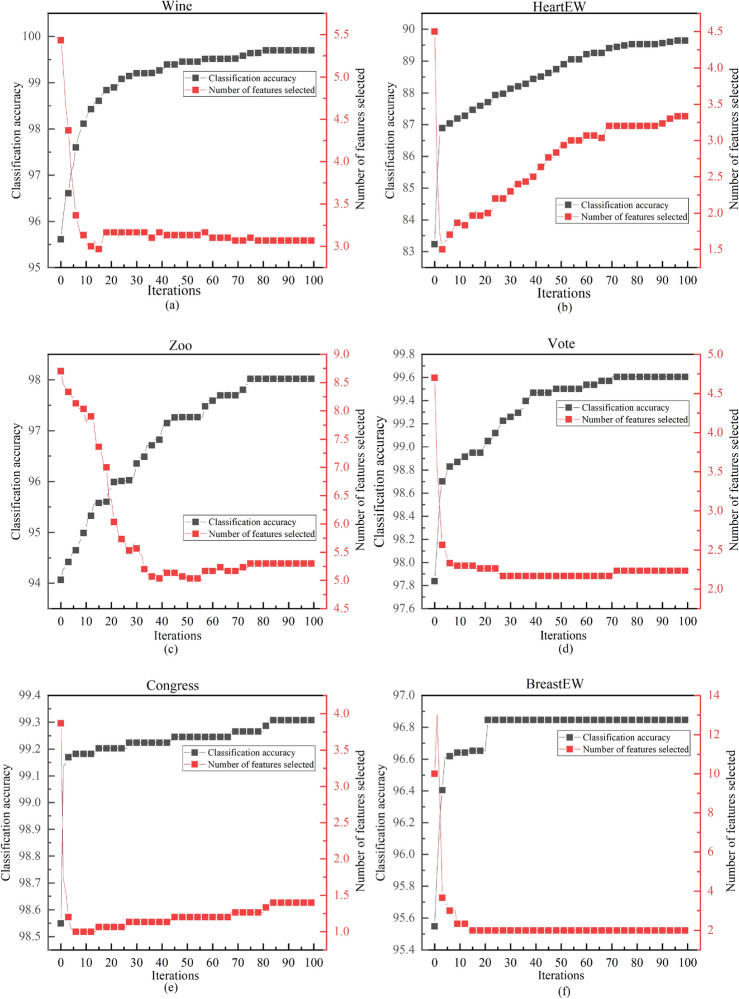
Classification accuracy versus the number of selected features process of ALI-CHoASH on UCI datasets.

**Figure 15:**
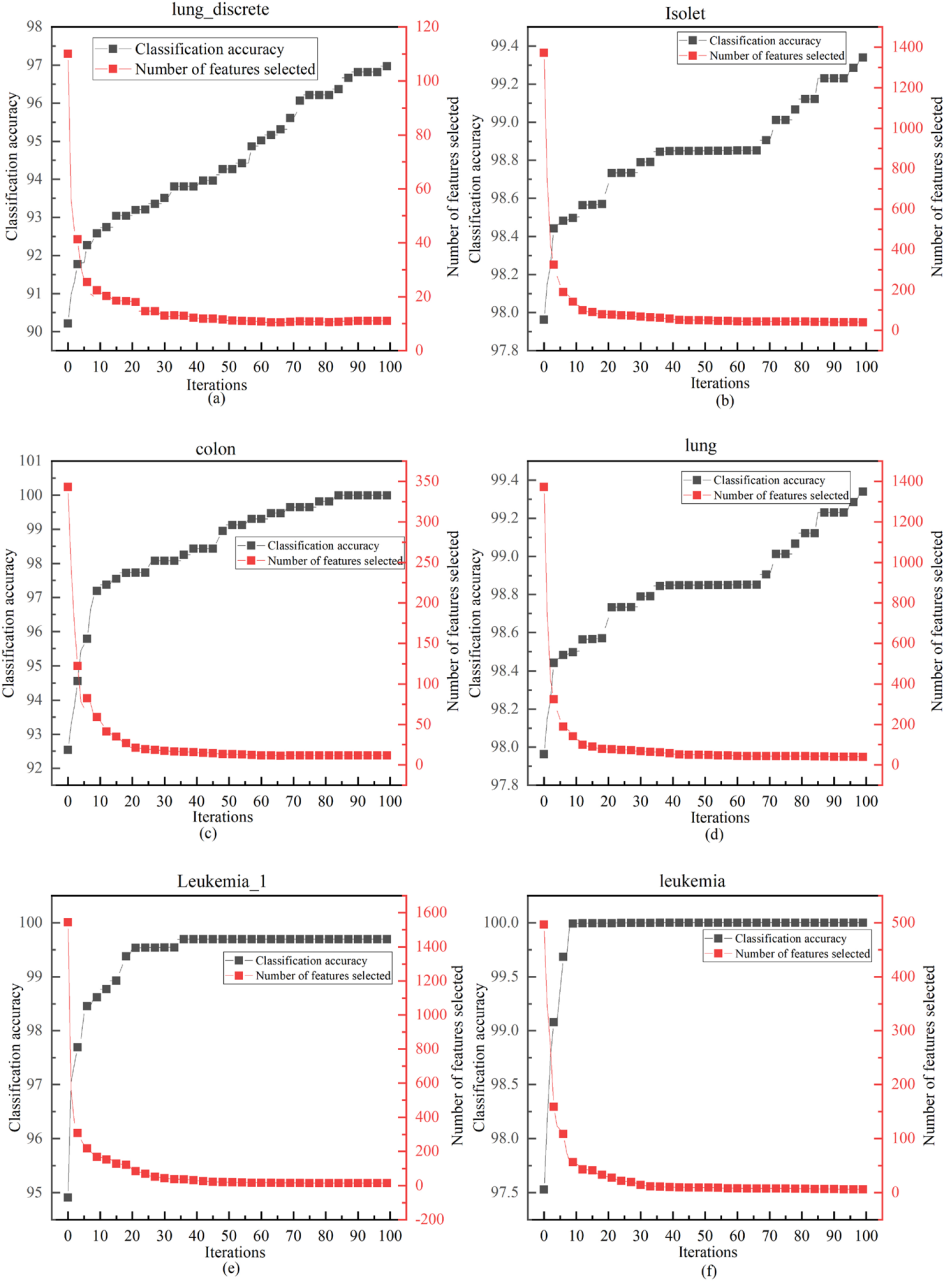
Classification accuracy versus the number of selected features process of ALI-CHoASH on ASU datasets.

**Figure 16:**
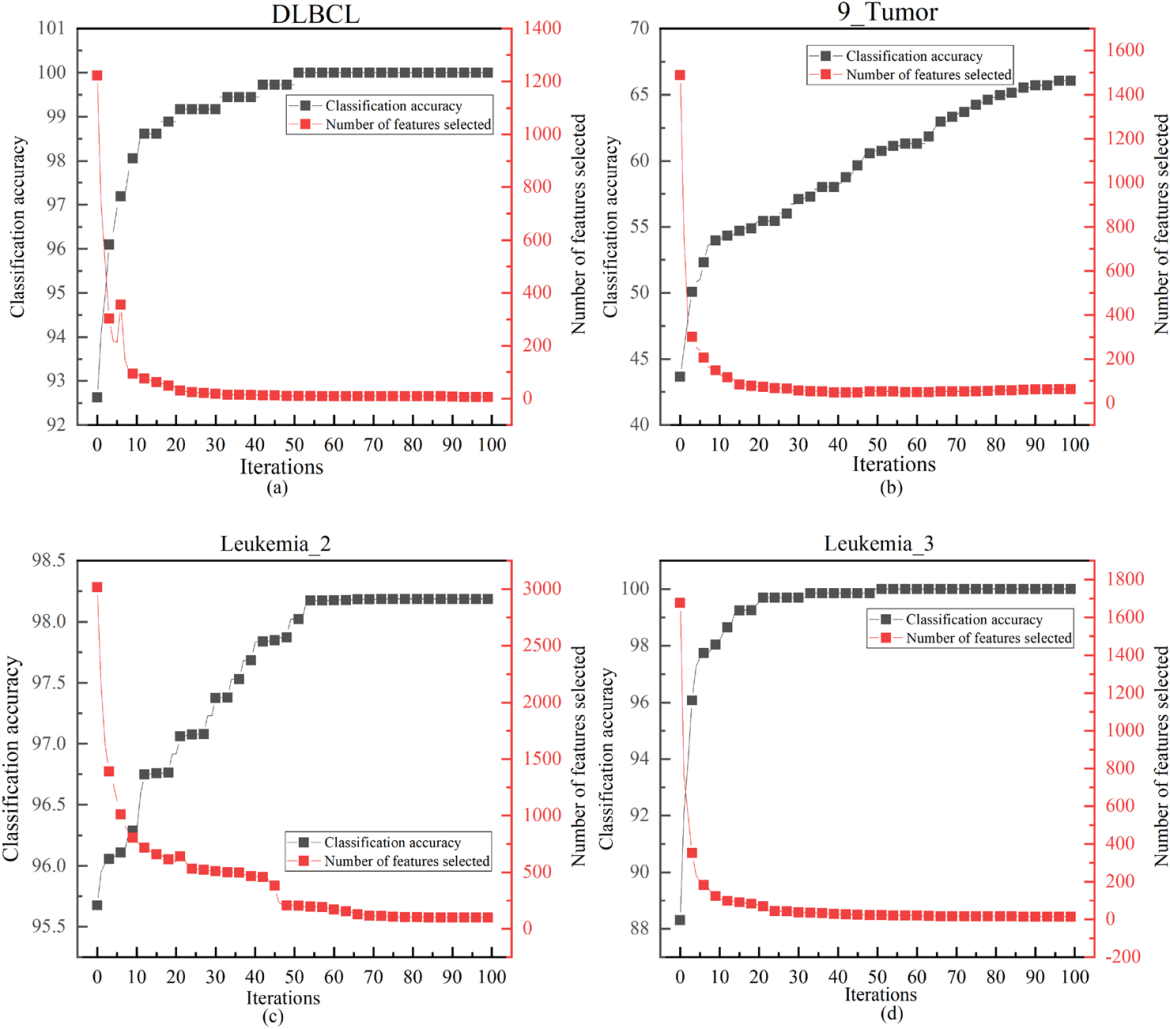
Classification accuracy versus the number of selected features process of ALI-CHoASH on gene datasets.

Figures 14, 15, and 16 show how the classification accuracy and the number of selected features change as the number of iterations increases. These figures show a similar trend, i.e., the accuracy of the classifier can be gradually improved by removing irrelevant or redundant features to the class labels on different test datasets. This suggests that as long as the selected subset of features contains enough information, better classification performance can be achieved than using all the features. The ALI-CHoASH method can improve classification accuracy while removing irrelevant or redundant features. In addition, a comparative analysis with Tables 1 and 3 shows that the ALI-CHoASH method only selects features between 0.13% and 28.57% of the original number of features, significantly reducing the number of original feature sets.

#### Comparison of classification performance of ALI-CHoASH with other heuristic algorithms

In the previous section, the proposed ALI-CHoASH algorithm performs well in feature selection. To better validate the effectiveness of the ALI-CHoASH method in feature selection, other heuristic algorithms are selected in this section to compare feature selection with the same evaluation criteria as in the previous experiments. Table 6 demonstrates the highest classification accuracy, lowest classification accuracy and variance based on the ALI-CHoASH algorithm with GMPBSA, SMA, GWO, BES, HHO and SSA algorithms in encapsulated feature selection. Meanwhile, Table 7 shows each algorithm’s average classification accuracy results. These comparisons provide further evidence of the superiority and effectiveness of the ALI-CHoASH algorithm in the feature selection problem.

**Table 6:** Classification accuracy for ALI-CHoASH and its meta-heuristic algorithm.

Data name	Indicators	ALI-CHoASH	GMPBSA	SMA	GWO	BES	HHO	SSA
Wine	Best	**100.00%**	98.15%	98.15%	98.15%	98.15%	98.15%	98.15%
	Worst	**98.15%**	96.30%	92.59%	90.74%	90.74%	87.04%	92.59%
	Std	3.32E−03	7.41E−03	1.61E−02	1.99E−02	1.77E−02	2.39E−02	1.68E−02
HeartEW	Best	**91.36%**	90.12%	87.65%	87.65%	87.65%	87.65%	87.65%
	Worst	**86.42%**	86.42%	81.48%	83.95%	81.48%	77.78%	77.78%
	Std	1.77E−02	1.02E−02	2.12E−02	7.60E−03	1.50E−02	2.81E−02	2.29E−02
Zoo	Best	**100.00%**	100.00%	100.00%	100.00%	100.00%	100.00%	100.00%
	worst	**96.67%**	96.67%	96.67%	96.67%	96.67%	93.33%	96.67%
	Std	1.67E−02	1.47E−02	1.24E−02	1.61E−02	8.31E−03	8.61E−03	1.33E−02
Vote	Best	**100.00%**	100.00%	94.44%	94.44%	94.44%	94.44%	94.44%
	Worst	**98.89%**	98.89%	91.11%	92.22%	92.22%	90.00%	92.22%
	Std	4.44E−03	1.99E−03	8.36E−03	6.87E−03	6.87E−03	1.18E−02	7.98E−−03
Congress	Best	**100.00%**	100.00%	98.47%	99.24%	99.24%	98.47%	99.24%
	Worst	**99.24%**	96.95%	96.95%	97.71%	97.71%	96.18%	97.71%
	Std	**3.05E−03**	7.02E−03	4.29E−03	4.62E−03	4.70E−03	6.46E−03	4.27E−03
BreastEW	Best	**96.49%**	95.32%	94.15%	94.15%	94.15%	94.15%	94.15%
	Worst	**94.74%**	93.57%	92.40%	90.64%	92.40%	91.81%	92.98%
	Std	5.09E−03	3.92E−03	2.92E−03	5.75E−03	3.29E−03	4.29E−03	2.78E−03
lung_discrete	Best	**100.00%**	95.45%	100.00%	100.00%	95.45%	100.00%	90.91%
	Worst	**95.45%**	90.91%	86.36%	90.91%	86.36%	81.82%	86.36%
	Std	2.14E−02	2.23E−02	4.35E−02	2.99E−02	2.27E−02	3.61E−02	1.82E−02
Isolet	Best	92.09%	89.10%	89.53%	**95.94%**	91.24%	90.81%	90.81%
	Worst	89.32%	86.75%	87.61%	**93.80%**	88.46%	88.68%	88.46%
	Std	7.48E−03	6.10E−03	4.92E−03	5.38E−03	7.29E−03	4.33E−03	6.18E−03
Colon	Best	**100.00%**	84.21%	100.00%	100.00%	100.00%	100.00%	78.95%
	Worst	**100.00%**	78.95%	89.47%	89.47%	84.21%	89.47%	73.68%
	Std	**0.00E+00**	1.79E−02	2.94E−02	3.68E−02	4.06E−02	3.02E−02	2.61E−02
Lung	Best	**100.00%**	98.36%	100.00%	100.00%	98.36%	100.00%	98.36%
	Worst	**98.36%**	96.72%	96.72%	98.36%	96.72%	96.72%	96.72%
	Std	8.03E−03	4.92E−03	8.39E−03	2.94E−03	2.94E−03	7.33E−03	6.56E−03
Leukemia_1	Best	**100.00%**	100.00%	100.00%	100.00%	100.00%	100.00%	100.00%
	Worst	95.45%	90.91%	90.91%	**100.00%**	95.45%	90.91%	90.91%
	Std	1.13E−02	2.61E−02	2.54E−02	0.00E+00	2.27E−02	3.39E−02	2.61E−02
DLBCL	Best	**100.00%**	87.50%	100.00%	100.00%	100.00%	100.00%	100.00%
	Worst	**100.00%**	1.76%	100.00%	100.00%	95.83%	95.83%	91.67%
	Std	**0.00E+00**	9.17E−01	0.00E+00	0.00E+00	7.48E−03	7.48E−03	2.28E−02
9_Tumor	Best	**83.33%**	55.56%	72.22%	77.78%	66.67%	66.67%	61.11%
	worst	**55.56%**	44.44%	50.00%	55.56%	50.00%	50.00%	50.00%
	Std	7.60E−02	3.65E−02	5.16E−02	5.64E−02	4.40E−02	5.35E−−02	3.56E−02
Leukemia	Best	**100.00%**	95.45%	100.00%	95.45%	100.00%	100.00%	86.36%
	Worst	**100.00%**	86.36%	90.91%	86.36%	86.36%	86.36%	77.27%
	Std	**0.00E+00**	2.33E−02	3.46E−02	2.06E−02	3.85E−02	4.25E−02	1.63E−02
Leukemia_2	Best	**100.00%**	90.91%	100.00%	95.45%	95.45%	95.45%	95.45%
	Worst	**95.45%**	81.82%	90.91%	95.45%	90.91%	90.91%	90.91%
	Std	2.23E−02	2.71E−02	1.43E−02	3.33E−16	1.82E−02	2.25E−02	2.08E−02
Leukemia_3	Best	**100.00%**	100.00%	100.00%	100.00%	100.00%	100.00%	100.00%
	Worst	**100.00%**	100.00%	95.45%	100.00%	100.00%	95.45%	95.45%
	Std	**0.00E+00**	0.00E+00	8.16E−03	0.00E+00	0.00E+00	8.16E−03	1.36E−02

Best value in each row of the table is identified in bold.

**Table 7:** Average classification accuracy for ALI-CHoASH and its meta-heuristic algorithm.

Data name	ALI-CHoASH (%)	GMPBSA (%)	SMA (%)	GWO (%)	BES (%)	HHO (%)	SSA (%)
Wine	**99.94**	97.78	96.11	96.67	95.49	94.75	95.93
HeartEW	**89.79**	88.02	86.21	87.37	86.79	83.66	85.88
Zoo	**98.33**	97.56	97.22	97.89	96.89	96.67	97.33
Vote	**99.78**	99.96	92.93	93.48	93.19	92.22	93.19
Congress	**99.39**	98.04	98.12	98.50	98.14	97.81	98.14
BreastEW	**95.96**	94.44	93.06	93.00	93.06	92.88	93.10
lung_discrete	**100**	93.64	90.30	95.61	91.52	90.76	90.00
Isolet	90.90	88.00	88.37	**94.84**	89.62	89.61	89.36
Colon	**100**	79.65	95.96	94.21	93.33	95.09	76.67
Lung	**99.34**	96.89	98.25	98.42	98.31	98.36	98.03
Leukemia_1	99.70	95.15	97.42	**100.00**	97.58	95.91	96.67
DLBCL	**100.00**	90.69	100.00	100.00	99.86	99.86	97.36
9_Tumor	65.74	49.81	57.04	**66.85**	54.44	57.04	53.89
Leukemia	**100.00**	87.58	96.52	94.70	91.52	96.21	81.52
Leukemia_2	**98.18**	84.85	95.61	95.45	94.55	93.48	92.27
Leukemia_3	**100.00**	100.00	99.85	100.00	100.00	99.85	99.55

Best value in each row of the table is identified in bold.

**Figure 17:**
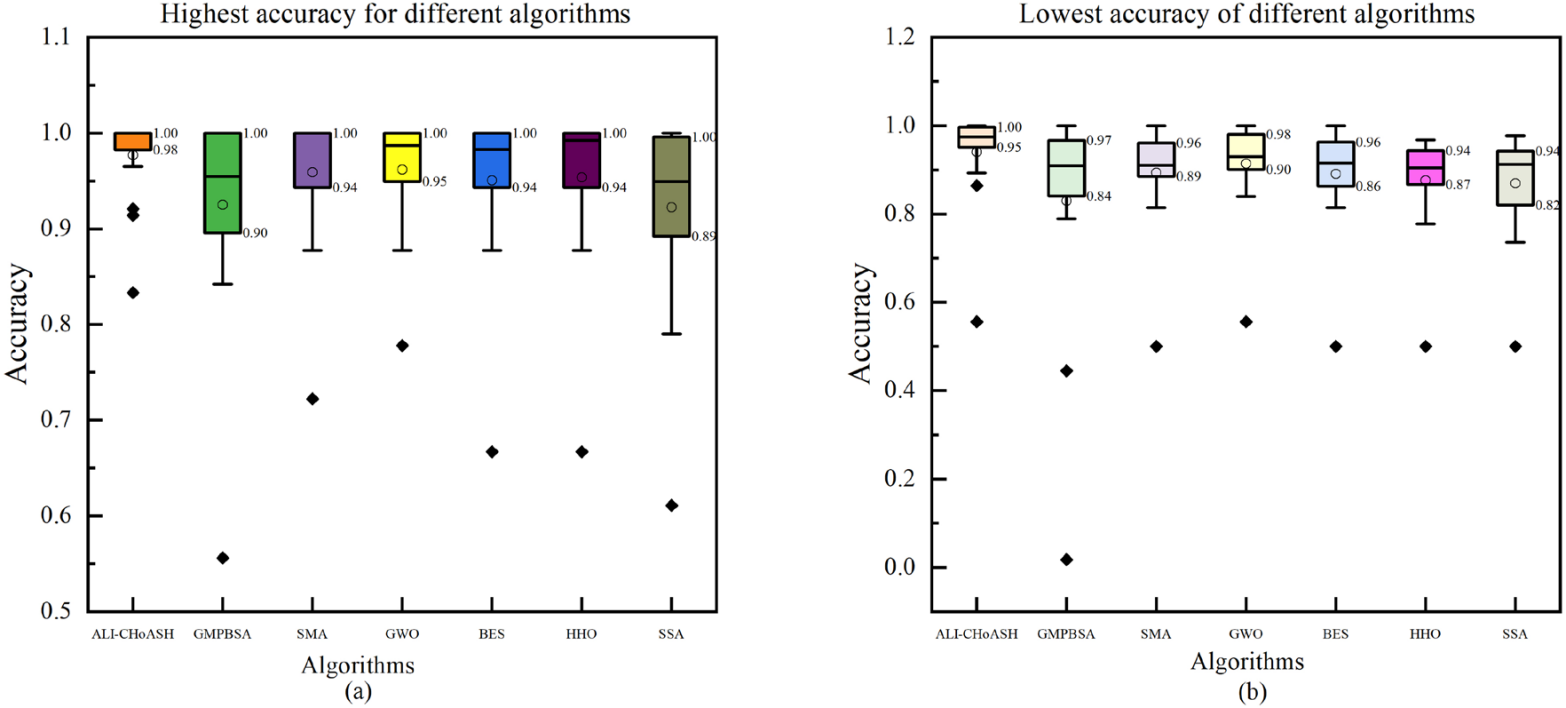
Comparison of classification accuracy of different algorithms.

As seen from Table 6, the highest classification accuracy achieved by ALI-CHoASH is in the leading position on 15 of the 16 datasets. It only slightly loses to GWO on the Isolet dataset, ranking second. Meanwhile, the lowest classification accuracy achieved by ALI-CHoASH is in the leading position on 15 datasets, losing only to GWO on the Isolet dataset, ranking second. To describe in more detail the differences between ALI-CHoASH and the other algorithms (GMPBSA, SMA, GWO, BES, HHO, and SSA), we can look at the comparison of the highest classification accuracies in Fig. 17a and the lowest classification accuracies in Fig. 17b from these graphs. We can see that the ALI-CHoASH algorithm performs optimally regarding classification effectiveness in terms of minimum, quartile (25th percentile), median, quartile (75th percentile) and maximum.

As can be seen from Table 7, the average classification accuracy achieved by ALI-CHoASH is in the leading position on 13 of the 16 datasets and only slightly loses to GWO on the Isolet, Leukemia_1 and 9_Tumor datasets, which is ranked second. Meanwhile, the average classification accuracies of the seven heuristic optimization algorithms, ALI-ChoASH, GMPBSA, SMA, GWO, BES, HHO and SSA, are 96.07%, 90.13%, 92.69%, 94.19%, 92.14%, 92.14% and 89.93%, respectively. It can be seen that the ALI-CHoASH algorithm has the best average classification accuracy. In addition, according to the statistical results in Table 7, it can be seen that the ALI-CHoASH algorithm has a significant advantage in the vast majority of datasets, winning the number of datasets with GMPBSA, SMA, GWO, BES, HHO, and SSA as 15, 15, 13, 15, 16, and 16, respectively.

#### Comparison of ALI-CHoASH performance with other heuristic algorithms for fitness values

To further demonstrate the effectiveness of the ALI-CHoASH algorithm, we compared it with six other optimization algorithms. The optimal fitness values of these seven algorithms are shown in Tables 8 and 9 shows the average fitness values of these seven algorithms. Firstly, as seen from Table 8, the optimal fitness values achieved by ALI-CHoASH lead on 13 of the 16 datasets, losing only slightly to GMPBSA on the Vote dataset, ranked second. It failed to SMA on the DLBCL dataset, ranking second and losing to GWO on the Leukemia_1 dataset, ranking second. Meanwhile, as can be seen from Table 8, the mean values of the optimal fitness of the seven heuristic optimization algorithms, namely ALI-ChoASH, GMPBSA, SMA, GWO, BES, HHO and SSA, are 4.23E-02, 1.02E-01, 7.36E-02, 5.99E-02, 8.00E-02, 8.01E-02, and 1.04E-01. It can be seen that the ALI-CHoASH algorithm has the best optimal fitness value. Finally, according to the statistical results in Table 8, it can be seen that the ALI-CHoASH algorithm has a significant advantage in the vast majority of datasets, winning the number of datasets with GMPBSA, SMA, GWO, BES, HHO, and SSA of 15, 15, 15, 16, 16, and 16, respectively. Where bold represents the optimal of the seven heuristic optimization algorithms under the dataset with the best fitness values.

**Table 8:** Optimal fitness values for ALI-CHoASH and its meta-heuristic algorithm.

Data name	ALI-CHoASH	GMPBSA	SMA	GWO	BES	HHO	SSA
Wine	**2.97E−03**	2.54E−02	4.12E−02	3.62E−02	4.81E−02	5.60E−02	4.42E−02
HeartEW	**1.04E−01**	1.22E−01	1.39E−01	1.28E−01	1.34E−01	1.65E−01	1.43E−01
Zoo	**1.98E−02**	2.90E−02	3.08E−02	2.41E−02	3.49E−02	3.75E−02	3.04E−02
Vote	1.13E−02	**4.97E−03**	7.26E−02	6.80E−02	7.31E−02	8.14E−02	7.24E−02
Congress	**6.92E−03**	2.26E−02	2.10E−02	1.75E−02	2.12E−02	2.50E−02	2.19E−02
BreastEW	**4.13E−02**	5.88E−02	6.99E−02	7.05E−02	7.07E−02	7.32E−02	7.17E−02
lung_discrete	**3.03E−02**	6.78E−02	9.63E−02	4.44E−02	8.57E−02	9.32E−02	1.03E−01
Isolet	**9.30E−02**	1.24E−01	1.19E−01	5.34E−02	1.08E−01	1.09E−01	1.10E−01
Colon	**5.80E−05**	2.06E−01	4.00E−02	5.82E−02	6.62E−02	4.88E−02	2.36E−01
Lung	**6.61E−03**	3.57E−02	1.74E−02	1.64E−02	1.75E−02	1.70E−02	2.43E−02
Leukemia_1	3.02E−03	5.29E−02	2.56E−02	**9.60E−04**	2.54E−02	4.19E−02	3.79E−02
DLBCL	1.13E−05	9.71E−02	**7.62E−06**	8.25E−04	1.84E−03	1.81E−03	3.10E−02
9_Tumor	**3.39E−01**	5.02E−01	4.25E−01	3.40E−01	4.54E−01	4.26E−01	4.61E−01
Leukemia	**9.05E−06**	1.28E−01	3.45E−02	5.35E−02	8.43E−02	3.76E−02	1.88E−01
Leukemia_2	**1.81E−02**	1.55E−01	4.35E−02	4.59E−02	5.52E−02	6.62E−02	8.14E−02
Leukemia_3	**1.33E−05**	4.93E−03	1.51E−03	8.76E−04	4.09E−04	2.13E−03	9.41E−03

Best value in each row of the table is identified in bold.

**Table 9:** Average fitness values for ALI-CHoASH and its meta-heuristic algorithm.

Data name	ALI-CHoASH	GMPBSA	SMA	GWO	BES	HHO	SSA
Wine	**8.65E−03**	3.23E−02	4.66E−02	3.73E−02	5.05E−02	6.09E−02	4.94E−02
HeartEW	**1.14E−01**	1.39E−01	1.46E−01	1.29E−01	1.37E−01	1.72E−01	1.50E−01
Zoo	**3.11E−02**	3.97E−02	3.31E−02	2.51E−02	3.62E−02	3.81E−02	3.21E−02
Vote	1.35E−02	**7.85E−03**	7.52E−02	6.89E−02	7.42E−02	8.26E−02	7.46E−02
Congress	**7.66E−03**	2.78E−02	2.21E−02	1.78E−02	2.20E−02	2.63E−02	2.31E−02
BreastEW	**4.39E−02**	6.27E−02	7.10E−02	7.08E−02	7.14E−02	7.42E−02	7.25E−02
lung_discrete	**5.51E−02**	7.80E−02	1.05E−01	6.00E−02	9.00E−02	9.90E−02	1.08E−01
Isolet	**1.07E−01**	1.27E−01	1.24E−01	7.59E−02	1.10E−01	1.13E−01	1.14E−01
Colon	**1.43E−02**	2.10E−01	5.42E−02	1.06E−01	8.58E−02	9.01E−02	2.42E−01
Lung	**1.15E−02**	3.57E−02	1.95E−02	1.78E−02	1.87E−02	1.89E−02	2.57E−02
Leukemia_1	**5.90E−03**	5.52E−02	3.42E−02	7.29E−03	2.93E−02	5.48E−02	4.46E−02
DLBCL	7.00E−03	9.98E−02	**1.34E−03**	4.43E−03	6.77E−03	9.17E−03	3.54E−02
9_Tumor	4.03E−01	5.06E−01	4.39E−01	**3.65E−01**	4.75E−01	4.59E−01	4.73E−01
Leukemia	**8.83E−04**	1.29E−01	4.98E−02	8.94E−02	1.05E−01	6.90E−02	1.92E−01
Leukemia_2	**2.39E−02**	1.55E−01	4.76E−02	4.89E−02	6.02E−02	7.48E−02	8.40E−02
Leukemia_3	6.02E−03	4.93E−03	**2.70E−03**	2.09E−03	1.34E−03	5.49E−03	1.06E−02

Best value in each row of the table is identified in bold.

Firstly, as seen from Table 9, the average fitness value achieved by ALI-CHoASH leads on 13 of the 16 datasets and only slightly loses to SMA and GWO on the DLBCL and 9_Tumor datasets, respectively, ranking third. It slightly loses to GMPBSA on the Vote dataset and ranks second. Secondly, as can be seen in Table 9, the average fitness values of the seven heuristic optimization algorithms, ALI-CHoASH, GMPBSA, SMA, GWO, BES, HHO and SSA, are 5.33E-02, 1.07E-01, 7.95E-02, 7.04E-02, 8.58E-02, 9.05E-02 and 1.08E- 01. it can be seen that the ALI-CHoASH algorithm has the best average fitness value. Finally, based on the statistics in Table 9, it is evident that the ALI-CHoASH algorithm has a significant advantage in the vast majority of datasets, winning the number of datasets with GMPBSA, SMA, GWO, BES, HHO, and SSA as 15, 14, 15, 16, 16, and 16, respectively.

As can be seen from Tables 3, 4, 5, 6, 7, 8 and 9 and Fig. 17, the ALI-CHoASH algorithm can handle the feature selection task well and find the optimal subset of features, resulting in satisfactory average classification accuracy.

#### Algorithm complexity analyses and comparisons

Time complexity is an important index to analyze the computational efficiency of the algorithm. Let the CHoA population size be *N*, the feature dimension be *D*, the maximum number of iterations be *T*, the time required to solve the value of the fitness function be $f\left( n \right)$, and the time to initialize the parameters is ${t_1}$. The standard CHoA time complexity available from the literature^21^ is:

40 $$\begin{aligned} T = \textrm{O}\left( {{t_1} + n + f\left( n \right) } \right) = \textrm{O}\left( {n + f\left( n \right) } \right) \end{aligned}$$

In the ALI-CHoASH algorithm proposed in this paper, the initial parameters of the algorithm, as well as the parameter setting time, are set to be consistent with CHoA. In addition, let the time for the chimpanzee social class multiple learning strategies be set to ${t_2}$, and the time for the improved lens imaging mapping strategy be ${t_3}$. The total time complexity of the ALI-CHoASH algorithm is:

41 $$\begin{aligned} {T_1} = \textrm{O}\left( {{t_1} + {t_2} + {t_3} + n + f\left( n \right) } \right) = \textrm{O}\left( {n + f\left( n \right) } \right) \end{aligned}$$

According to the above analysis, this paper proposes a series of improvement strategies for the shortcomings of the standard CHoA, and these improvement strategies do not increase the algorithm’s time complexity and do not affect the execution efficiency of the algorithm. The comparative analysis of the average running time of the seven heuristic optimization algorithms in Table 10 shows that the ALI-CHoASH algorithm has the longest running time. Although ALI-CHoASH effectively improves the convergence speed of the algorithm by ensuring population diversity through a multi-learning strategy and using an improved lens imaging mapping strategy, it still faces the problem of high computational cost. Therefore, future research must explore obtaining a subset of features with strong discriminative ability in a shorter time.

**Table 10:** The running time (/s) for ALI-CHoASH and its meta-heuristic algorithm.

Data name	ALI-CHoASH	GMPBSA	SMA	GWO	BES	HHO	SSA
Wine	11.65	7.72	2.62	7.62	3.3	5.83	3.78
HeartEW	14.88	10.08	3.09	10.03	4.39	7.44	5.04
Zoo	8.52	5.65	2.35	5.56	2.4	4.29	2.7
Vote	18.45	11.37	3.45	10.9	5.15	8.52	5.57
Congress	22.02	15.87	4.56	14.79	6.42	11.58	7.63
BreastEW	27.99	19.22	5.54	17.98	6.85	14.12	9.55
lung_discrete	17.4	5.07	6.25	8.34	2.29	4.71	2.98
Isolet	165.6	84.89	46.86	92.63	42.6	78.52	48.13
Colon	65.41	11.4	27.34	31.15	4.98	12.6	8.66
Lung	63.3	26.76	45.55	56.11	10.32	24.69	18.65
Leukemia_1	178.52	23.78	66.86	75.96	11.48	29.69	19.26
DLBCL	165.78	24.23	67.6	77.99	11.35	30.03	19.73
9_Tumor	439.09	284.53	71.54	81.55	12.97	19.63	20
Leukemia	234.9	30.04	86.81	99.05	13.96	36.81	24.65
Leukemia_2	224.38	30.8	90.55	103.52	15.08	40.01	25.49
Leukemia_3	363.89	45.09	180.8	150.94	159.19	21.85	59.05

#### Analysis of convergence curves

**Figure 18:**
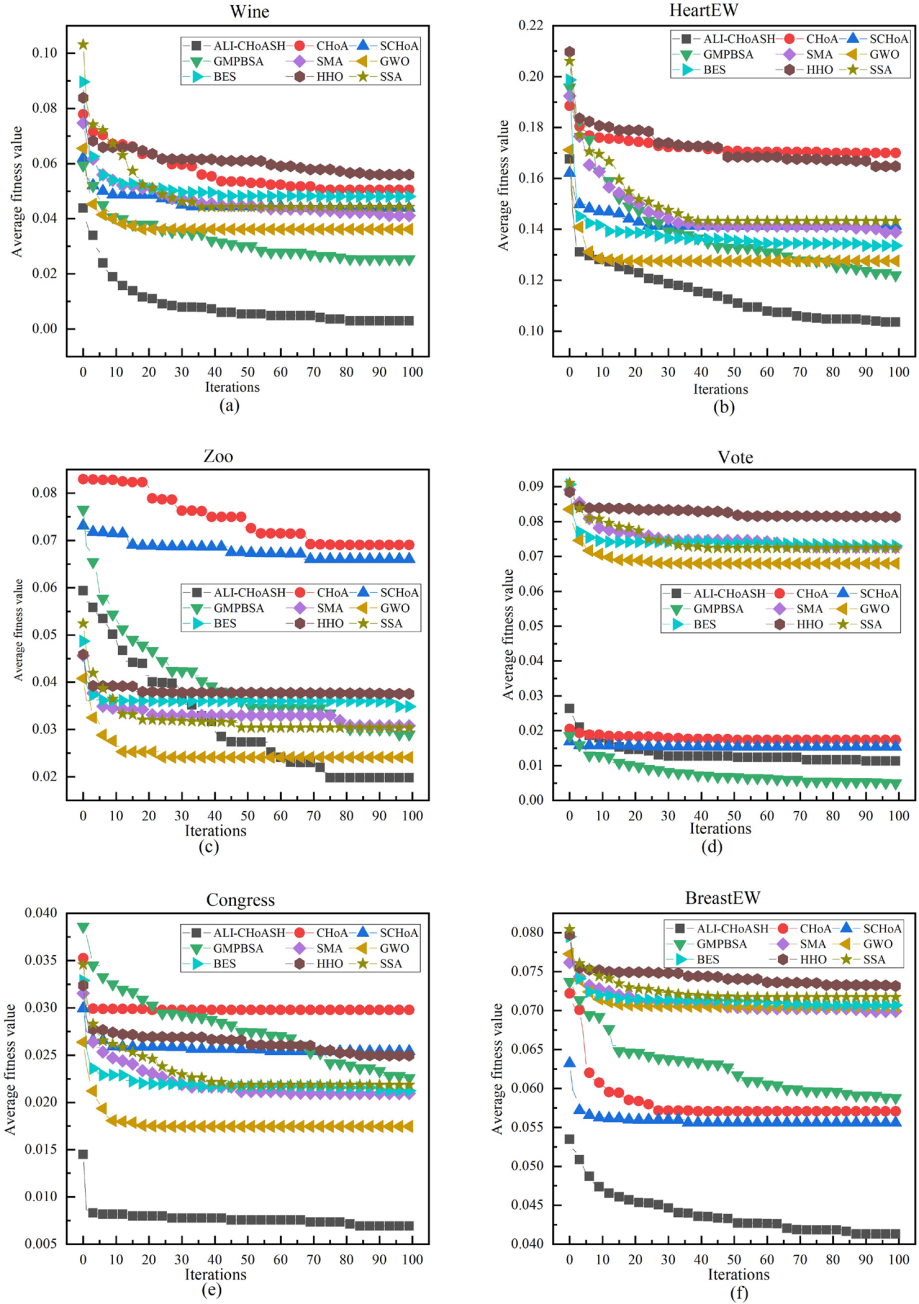
Convergence curves of all algorithms on UCI datasets.

**Figure 19:**
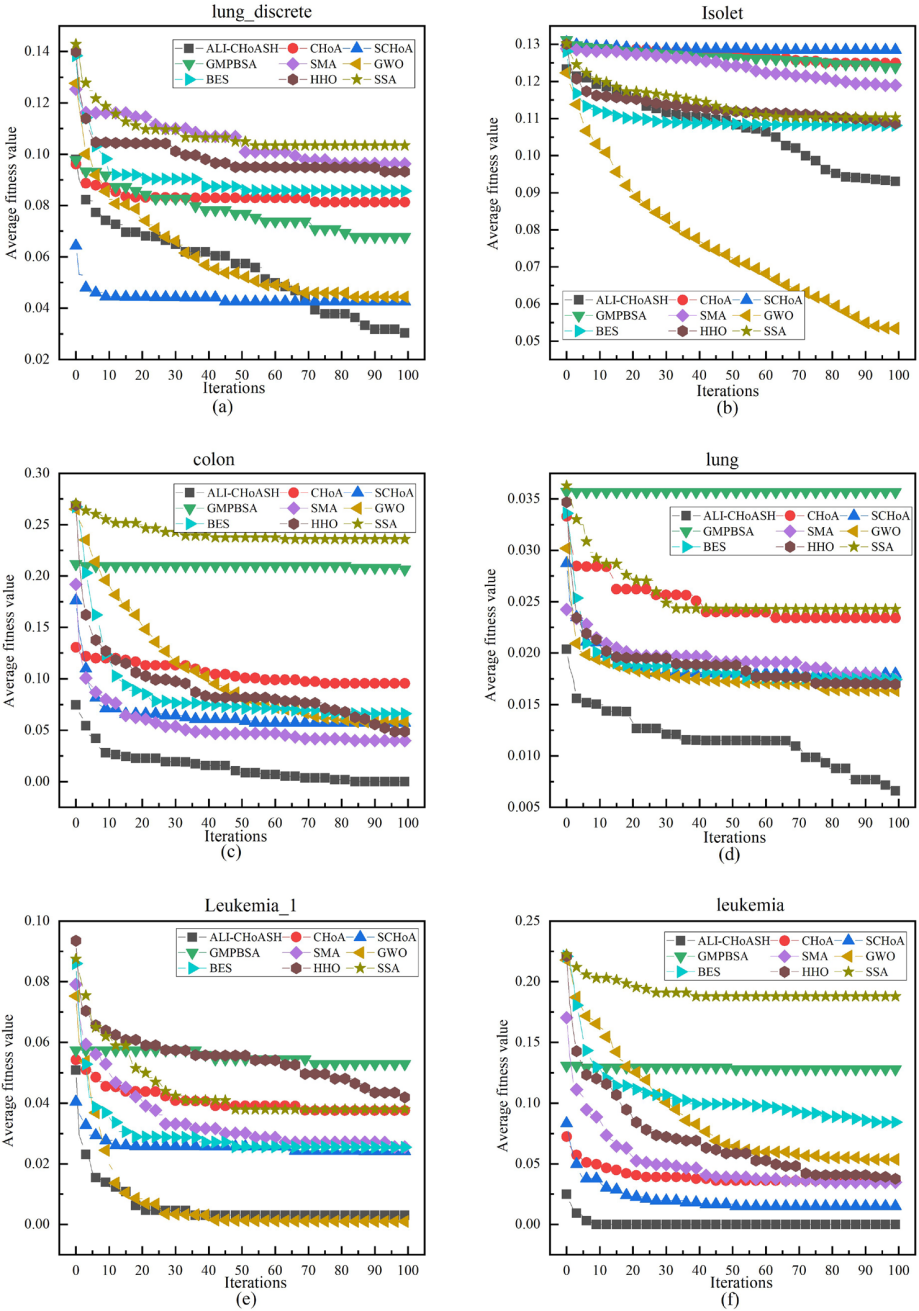
Convergence curves of all algorithms on ASU datasets.

**Figure 20:**
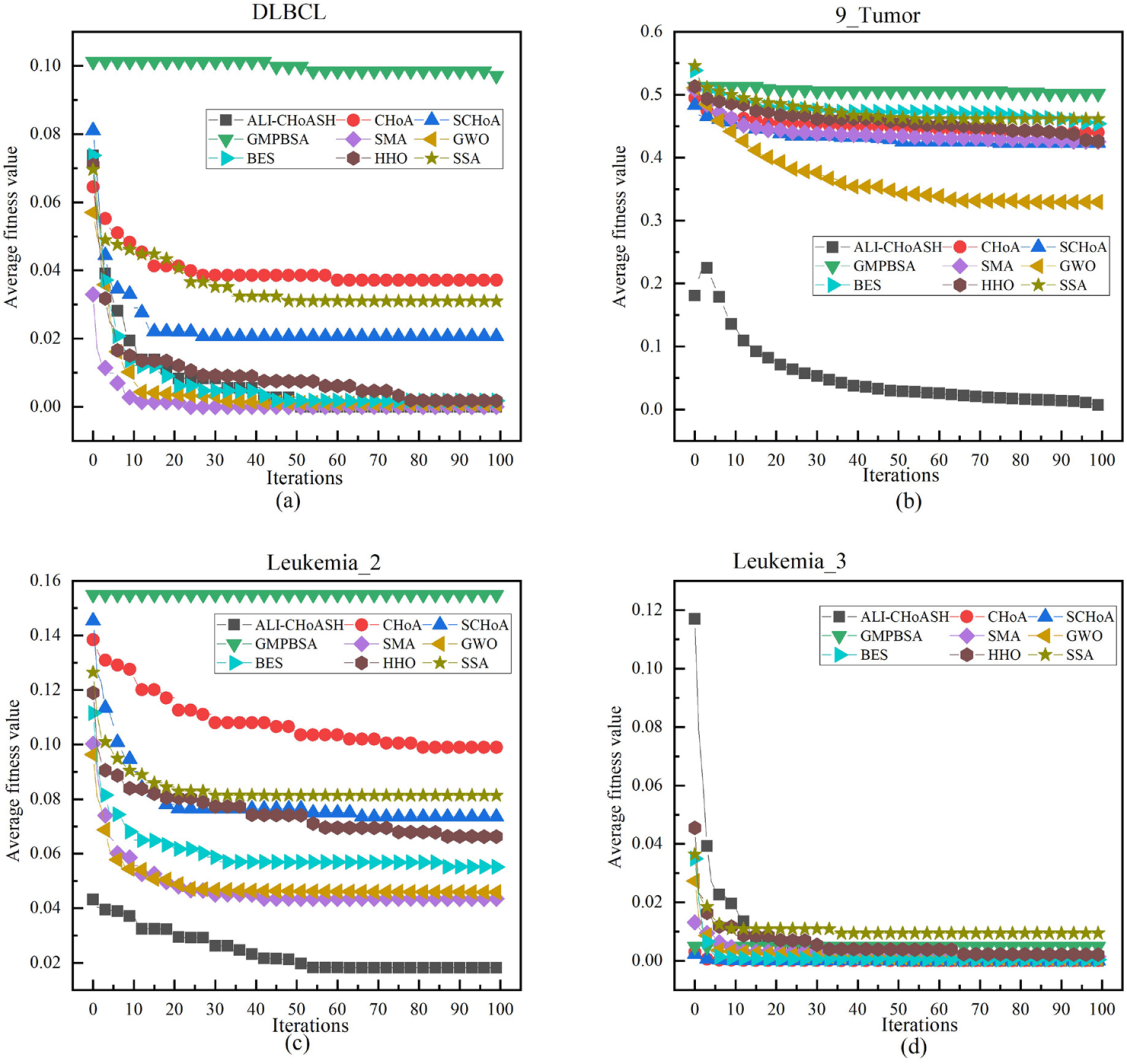
Convergence curves of all algorithms on gene datasets.

Since the goal of the feature selection process is to minimize the fitness function value, the smaller the fitness function value, the better the convergence performance of the corresponding algorithm. To further compare the convergence performance of the ALI-CHoASH algorithm, Figs. 18, 19 and 20 show the fitness convergence curves of ALI-CHoASH with the heuristic feature selection algorithms such as CHoA, SCHoA, GMPBSA, SMA, GWO, BES, HHO, and SSA on 16 datasets. Meanwhile, this section observes and judges the performance advantages and disadvantages of the algorithms by analyzing the convergence curves of the algorithms and further observes the convergence speed of the algorithms through the convergence curves. Figures 18, 19 and 20 show the comparison graphs of convergence curves of different algorithms on low-dimensional and high-dimensional datasets. From Figs. 18, 19 and 20, it can be seen that in Figs. 18a–c, e,f, 19a–d and 20a–d, the convergence speed of ALI-ChoASH is faster than the other eight algorithms throughout the entire iteration process, and the convergence accuracy is the best among these eight algorithms. This indicates that the ALI-CHoASH algorithm is significantly better than the other heuristic algorithms.

As can be seen from Figs. 18, 19 and 20, the ALI-CHoASH algorithm has faster convergence on 12 of the 16 test datasets (Wine, HeartEW, Zoo, Congress, BreastEW, lung_discrete, colon, lung, 9_Tumor, leukemia, Leukemia_2 and Leukemia_3) have faster convergence. For the remaining four test datasets (Vote, Isolet, DLBCL and Leukemia_1), the ALI-CHoASH algorithm also shows better convergence performance than most of the compared algorithms. This further indicates that the mechanism designed in the ALI-CHoASH algorithm can effectively improve the algorithm’s search capability, which can find a higher-quality subset of features in a limited number of iterations. The results in Tables 3, 8 and 9 also demonstrate the effectiveness of the ALI-CHoASH algorithm in searching the high-dimensional feature space.

**Figure 21:**
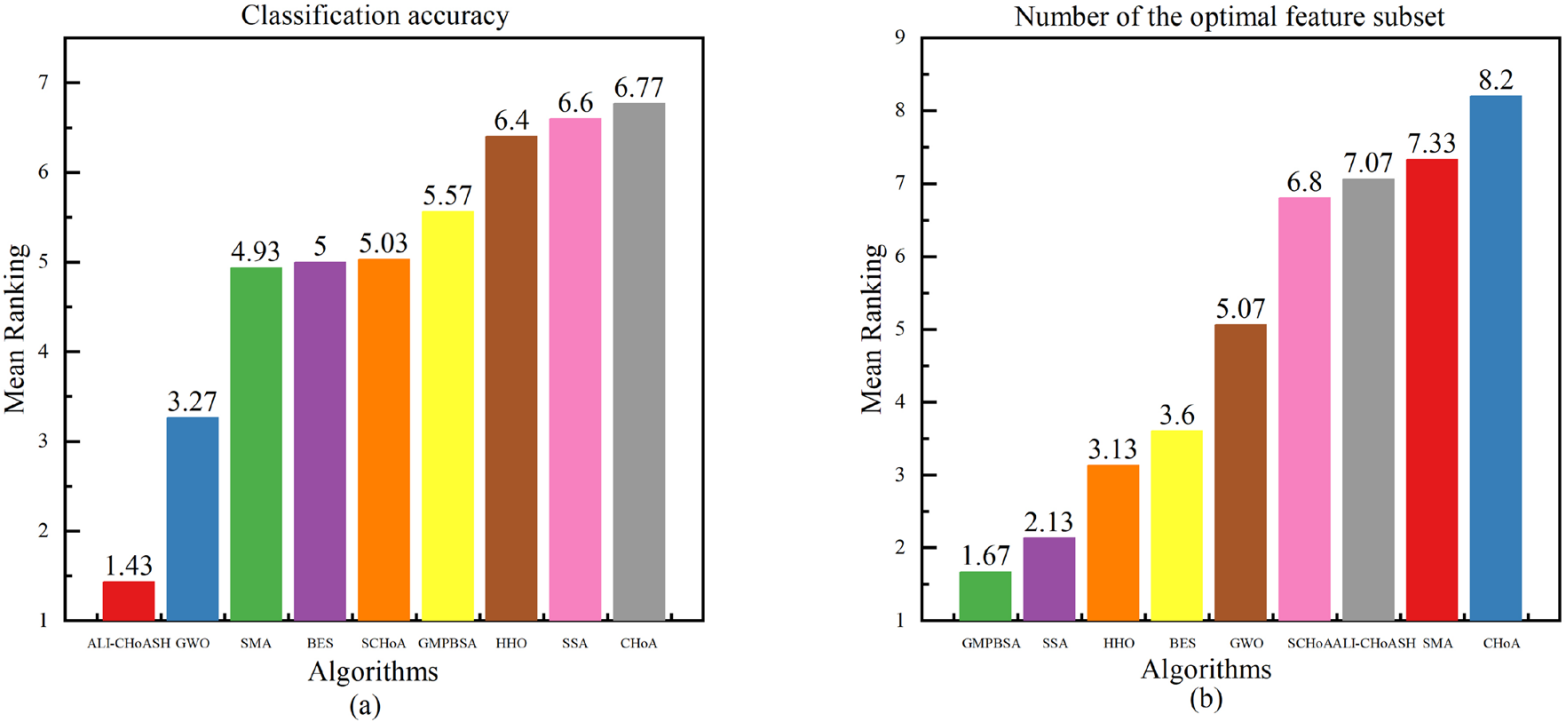
Mean Friedman test ranks of nine algorithms on sixteen datasets.

Figure 21 shows the classification accuracy and the optimal number of feature subsets based on the average results of the Friedman ranking test for nine algorithms on sixteen datasets.

As shown in Fig. 21a for classification accuracy, the ALI-CHoASH ranks first, followed by the GWO, SMA, BES, SCHoA, GMPBSA, HHO, SSA, and CHoA algorithms. As shown in Fig. 21b for the optimal number of feature subsets, the GMPBSA ranks first, followed by the SSA, HHO, BES, GWO, SCHoA, ALI-CHoASH, SMA, and CHoA algorithms.

In summary, regarding the feature selection process, the proposed improved mechanism of the ALI-CHoASH method can effectively improve the classification accuracy and reduce the dimensionality of the selected data features in sample data of different dimensions and capacities. Meanwhile, the technique performs better classification in the feature selection task, successfully selecting features with discriminative solid ability. Its solution fitness value, convergence speed and stability are better than CHoA, SCHoA, GMPBSA, SMA, GWO, BES, HHO and SSA. Therefore, the ALI-CHoASH algorithm has a better overall optimization finding ability and higher stability than other compared algorithms.

#### Wilcoxon rank-sum test

To verify the effectiveness and stability of the ALI-CHoASH algorithm. In this section, the Wilcoxon rank sum test is used to confirm whether there is a significant difference in the running results between this algorithm and other algorithms. Therefore, the results of 9 algorithms tested independently 30 times on 16 test data are taken as samples. $p < 5\%$ indicates significant variability between the two algorithms compared. When $p \ge 5\%$, it suggests that the optimality finding results of the two algorithms under comparison are the same. The comparison of ALI-CHoASH with CHoA, SCHoA, GMPBSA, SMA, GWO, BES, HHO and SSA is denoted as P1, P2, P3, P4, P5, P6, P7, and P8, respectively. Table 11 compares ALI-CHoASH with CHoA, SCHoA, GMPBSA, and SMA under 16 test data sets. GWO, BES, HHO and SSA values were calculated in the rank sum test. As can be seen from the analysis in Table 11, the values are much less than 5% in the vast majority of the test datasets. Among them, on the Zoo dataset, the results of the ALI-CHoASH and SSA algorithms for finding the best are the same on the whole. On the DLBCL dataset, the optimization results of ALI-CHoASH and GWO algorithms are the same overall. On the Leukemia_1 dataset, the optimization results of the ALI-CHoASH and SMA algorithms are the same general.

**Table 11:** Results of Wilcoxon rank sum test.

Data name	P1	P2	P3	P4	P5	P6	P7	P8
Wine	3.52E−18	3.54E−18	3.84E−18	3.52E−18	3.72E−18	3.53E−18	3.57E−18	3.52E−18
HeartEW	3.85E−18	3.95E−18	3.89E−18	3.87E−18	3.83E−18	3.83E−18	3.80E−18	3.83E−18
Zoo	3.52E−18	3.36E−18	3.88E−18	3.74E−02	1.20E−05	3.60E−05	2.62E−08	**4.16E−01**
Vote	2.71E−16	5.84E−10	3.85E−18	3.66E−18	3.33E−18	3.55E−18	3.56E−18	3.35E−18
Congress	3.03E−18	3.23E−18	3.88E−18	3.51E−18	3.06E−18	3.41E−18	3.75E−18	3.20E−18
BreastEW	3.73E−18	3.73E−18	3.87E−18	3.79E−18	3.73E−18	3.82E−18	3.88E−18	3.73E−18
lung_discrete	3.97E−18	5.62E−10	3.97E−18	3.85E−18	9.19E−08	3.86E−18	3.87E−18	3.84E−18
Isolet	3.89E−18	3.89E−18	3.89E−18	3.89E−18	3.90E−18	3.14E−02	2.68E−12	3.90E−18
Colon	3.49E−18	3.65E−18	3.77E−18	3.37E−18	3.89E−18	3.63E−18	3.82E−18	3.71E−18
Lung	3.71E−18	3.72E−18	3.78E−18	3.77E−18	3.89E−18	3.85E−18	3.84E−18	3.68E−18
Leukemia_1	3.63E−18	3.54E−18	3.23E−18	3.32E−18	2.19E−02	3.63E−18	3.87E−18	2.37E−18
DLBCL	4.27E−18	3.91E−18	3.81E−18	3.87E−18	8.00E−06	**4.77E−01**	3.99E−08	4.30E−18
9_Tumor	3.90E−18	3.90E−18	3.90E−18	3.90E−18	3.90E−18	3.90E−18	3.90E−18	3.90E−18
Leukemia	3.78E−18	3.78E−18	3.78E−18	3.83E−18	3.90E−18	3.88E−18	3.88E−18	3.85E−18
Leukemia_2	3.61E−18	3.46E−18	3.46E−18	3.46E−18	3.85E−18	3.79E−18	3.87E−18	3.46E−18
Leukemia_3	3.70E−05	3.70E−05	1.03E−04	1.70E−02	**2.98E−01**	3.70E−05	2.00E−05	3.69E−08

Best value in each row of the table is identified in bold.

Table 11 shows an overall significant difference between ALI−CHoASH and the other eight algorithms, thus indicating that ALI-CHoASH possesses better effectiveness than the different algorithms.

## Conclusion

The presence of irrelevant and redundant features in high-dimensional data increases the machine learning model’s time and space complexity, thus seriously affecting the accuracy and operational efficiency. The traditional chimpanzee optimization algorithm is prone to problems such as slow convergence speed and low optimization search accuracy, leading to the inability to remove irrelevant and redundant features effectively. To balance the ability of local exploration and global exploitation and avoid local optimality. In this paper, we conduct an in-depth study of the chimp population hierarchy, propose the enhanced chimp hierarchy optimization algorithm for adaptive lens imaging (ALI-CHoASH), and incorporate this algorithm into the feature selection algorithm. The following conclusions are drawn by combining the exploration and exploitation capacity percentage, classification accuracy, average optimal fitness value and optimal fitness value:

- Individual chimp inter-somatic relationships were optimized by designing a chimp social hierarchy. The social hierarchy factor was used to control the hunting patterns of chimp groups and adjust the balance between local exploration and global exploitation, guiding individual chimps to search more broadly within their social hierarchy.
- In the late iteration, due to the decline of population diversity, the traditional CHoA algorithm can easily fall into the local optimum. The position of individual chimps is optimised using the oppositional learning strategy of adaptive lens imaging, which improves the ability to jump out of the local optimum solution in the late iteration.
- Comparison test experiments regarding exploration and exploitation capacity percentage, classification accuracy and optimal fitness value show that the ALI-CHoASH algorithm has a better convergence effect and optimisation accuracy, proving that the improvement strategy proposed in this paper is effective.

In conclusion, ALI-CHoASH has some advantages in addressing feature selection. However, it still has shortcomings in reducing the feature dimensions of datasets such as Isolet, Leukemia_1 and 9_Tumor. Therefore, in future work, how to optimize the chimpanzee social hierarchy and hunting patterns, refine the classification optimization ability of ALI-CHoASH, and improve the classification effect of the algorithm on higher feature dimensions will be the main focus of future research.

## Data Availability

The experimental data set selects the world-famous data set (https://archive.ics.uci.edu/ , https://ckzixf.github.io/dataset.html and https://jundongl.github.io/scikit-feature/datasets.html).
